# Development and Testing of a Spray-Dried Tuberculosis Vaccine Candidate in a Mouse Model

**DOI:** 10.3389/fphar.2021.799034

**Published:** 2022-01-21

**Authors:** Mellissa Gomez, Mushtaq Ahmed, Shibali Das, Joseph McCollum, Leah Mellett, Rosemary Swanson, Ananya Gupta, Nicholas B. Carrigy, Hui Wang, David Barona, Shital Bachchhav, Alana Gerhardt, Chris Press, Michelle C. Archer, Hong Liang, Emilie Seydoux, Ryan M. Kramer, Philip J. Kuehl, Reinhard Vehring, Shabaana A. Khader, Christopher B. Fox

**Affiliations:** ^1^ Department of Mechanical Engineering, University of Alberta, Edmonton, AB, Canada; ^2^ Department of Molecular Microbiology, Washington University in St. Louis, School of Medicine, St. Louis, MO, United States; ^3^ Infectious Disease Research Institute, Seattle, WA, United States; ^4^ Lovelace Biomedical, Albuquerque, NM, United States; ^5^ Department of Global Health, University of Washington, Seattle, WA, United States

**Keywords:** dry powder vaccine, respiratory delivery, in vivo murine model, nose-only inhalation device, particle engineering, vaccine adjuvant formulation, tuberculosis, ID93+GLA-SE

## Abstract

Converting a vaccine into a thermostable dry powder is advantageous as it reduces the resource burden linked with the cold chain and provides flexibility in dosage and administration through different routes. Such a dry powder presentation may be especially useful in the development of a vaccine towards the respiratory infectious disease tuberculosis (TB). This study assesses the immunogenicity and protective efficacy of spray-dried ID93+GLA-SE, a promising TB vaccine candidate, against *Mycobacterium tuberculosis (Mtb)* in a murine model when administered via different routes. Four administration routes for the spray-dried ID93+GLA-SE were evaluated along with relevant controls—1) reconstitution and intramuscular injection, 2) reconstitution and intranasal delivery, 3) nasal dry powder delivery via inhalation, and 4) pulmonary dry powder delivery via inhalation. Dry powder intranasal and pulmonary delivery was achieved using a custom nose-only inhalation device, and optimization using representative vaccine-free powder demonstrated that approximately 10 and 44% of the maximum possible delivered dose would be delivered for intranasal delivery and pulmonary delivery, respectively. Spray-dried powder was engineered according to the different administration routes including maintaining approximately equivalent delivered doses of ID93 and GLA. Vaccine properties of the different spray-dried lots were assessed for quality control in terms of nanoemulsion droplet diameter, polydispersity index, adjuvant content, and antigen content. Our results using the *Mtb* mouse challenge model show that both intranasal reconstituted vaccine delivery as well as pulmonary dry powder vaccine delivery resulted in *Mtb* control in infected mice comparable to traditional intramuscular delivery. Improved protection in these two vaccinated groups over their respective control groups coincided with the presence of cytokine-producing T cell responses. In summary, our results provide novel vaccine formulations and delivery routes that can be harnessed to provide protection against *Mtb* infection.

## Introduction

Tuberculosis (TB) is a highly infectious respiratory disease that was responsible for the deaths of 1.2 million people worldwide in 2019 (WHO, 2020). There has been a rise in drug-resistant strains, with approximately 500,000 people developing drug-resistant TB in 2019 (WHO, 2020). The increased risk posed by these strains illustrates the need for effective prevention programs. However, the Bacille Calmette-Guerin (BCG) vaccine, the only licensed TB vaccine, provides variable efficacy in preventing TB in adults (Dockrell and Smith, 2017; WHO, 2020). Thus, several new TB vaccine candidates have been investigated as alternatives. For instance, the M72/AS01E TB vaccine consisting of a recombinant fusion protein antigen with a liposome adjuvant system containing a naturally derived Toll-like receptor 4 ligand and a saponin reduced disease progression after 3 years by 49.7% in Phase 2 clinical testing (Tait et al., 2019). Another promising candidate is the ID93+GLA-SE vaccine developed by the Infectious Disease Research Institute. ID93+GLA-SE is a subunit vaccine that consists of an antigen, ID93, and an adjuvant system, GLA-SE, that consists of a synthetic Toll-like receptor 4 ligand formulated in a nanoemulsion (Bertholet et al., 2010; Coler et al., 2018; Penn-Nicholson et al., 2018). The ID93+GLA-SE vaccine candidate is currently undergoing Phase II clinical trials as a liquid injectable presentation (ClinicalTrials.gov, 2019; Day et al., 2021). Like many other vaccines, the liquid dosage form requires refrigeration to maintain potency, and therefore widespread global vaccine rollouts may be hindered by the resource burden associated with maintaining the cold chain. Conversion of a liquid product into a thermostable dry form may improve distribution. Previously, we developed a thermostable lyophilized presentation of ID93+GLA-SE designed for reconstitution prior to injection, and it is currently undergoing Phase 1 clinical evaluation (Kramer et al., 2018). Another method of desiccation is through spray drying, wherein an atomized liquid product is dried into a powder. Spray drying has been shown to successfully confer thermostability to several experimental vaccines (Kanojia et al., 2018; LeClair et al., 2019) and approved vaccines (Kunda et al., 2019; Price et al., 2020).

Spray drying the ID93+GLA-SE into a thermostable dry powder form has been explored previously. Initially, the ID93+GLA-SE formulation was spray-dried into a powder designed for eventual reconstitution using the disaccharide trehalose as a stabilizing excipient (Gomez et al., 2021a). This presentation demonstrated promising long-term room temperature stability and short-term high temperature stability. While parenteral injection is the most common method of administration for vaccines, administration through inhalation has been gathering more attention. Inhalable routes allow for high-dose targeting at the site of infection while minimizing possible systemic toxic effects (Hickey et al., 2016). Additionally, immunization studies on mice and nonhuman primates have shown that administration via intranasal (IN) and pulmonary routes conferred greater protection against *Mtb* and other respiratory infections, at least in part due to induction of more effective mucosal immune responses (Derrick et al., 2014; Griffiths et al., 2016a; Ahmed et al., 2017a; Ahmed et al., 2017b; Raeven et al., 2018; Vierboom et al., 2021). Given the potential benefits of respiratory administration, an inhalable presentation of the spray-dried ID93+GLA-SE powder designed for pulmonary delivery was developed (Gomez et al., 2021b). The study established a lead inhalable excipient system consisting of trehalose and small amounts of the tripeptide trileucine. The spray-dried inhalable presentation of ID93+GLA-SE showed promising thermostability over 1 year of storage (Gomez et al., 2021c). While these results are encouraging, *in vivo* preclinical testing is necessary to assess safety and immunogenicity prior to testing with humans.

Mice are commonly used for preclinical trials because of their small size, low maintenance cost, and short growth time, all of which allow for many to be tested at one time for statistical validity (Nadithe et al., 2003; Cryan et al., 2007). Several methods of aerosol delivery have been developed expressly for the murine respiratory system as it is often not feasible to deliver aerosols using human clinical inhalation devices. For example, nose-only exposure systems, wherein mice are restrained in tubes against noseports, have been successfully used in rodent inhalation studies (Wang et al., 2014; Verco et al., 2018; Carrigy et al., 2019). These aerosol delivery systems are generally comprised of two main components: a device to aerosolize the liquid or dry product, and a nose-only exposure system to restrain the mice. The aerosolization of dry products through dispersion of a dry powder can be accomplished through several methods, such as the use of a dust generator (Wang et al., 2014; Chand et al., 2016; Cosnier et al., 2016). Spray drying of ID93+GLA-SE has shown promising results as a thermostable and inhalable presentation. However, previous work evaluated the spray-dried product through biochemical assays and not with an *in vivo* model. Furthermore, these studies did not test for the optimal method of administration. In the present study, administration of spray-dried ID93+GLA-SE through different routes was evaluated in a murine model, including intramuscular injection, intranasal liquid delivery, intranasal powder delivery, and pulmonary powder delivery. Particle engineering was used to design powder suitable for each method of administration. Powder inhalation was completed using a custom aerosol delivery device. This device was characterized and optimized prior to conducting the study. The murine studies consisted of immunogenicity studies comparing the immune response induced by the different routes and protective efficacy studies wherein the mice were challenged with *Mycobacterium tuberculosis* (*Mtb*) after immunization and bacterial burden was compared for the different routes.

## Materials and Methods

### Mouse Model Experimental Matrix

The experimental matrix, shown in Table 1, was designed to assess the viability of intranasal and pulmonary routes of administration of the spray-dried version of the ID93+GLA-SE vaccine. The dosing target was 0.4 µg of ID93 antigen and 1 µg of GLA adjuvant delivered to each mouse. The first group of mice was immunized with the reconstituted vaccine via intramuscular injection as a positive control. The remaining groups assessed delivery of the reconstituted formulation to the nose [liquid intranasal administration (Group 3)], delivery of the dry powder formulation targeting deposition in the nose [dry powder intranasal administration (Group 5)], and delivery of the dry powder formulation targeting deposition in the nose and lungs [dry powder pulmonary administration (Group 7)]. Isolated delivery to the lungs is not possible using a nose-only exposure system given that mice are obligate nose breathers.

**TABLE 1:** Experimental matrix for the assessment of administration route of spray-dried ID93+GLA-SE forms in mice. Nomenclature: V–Vaccine; A–Adjuvant; L–Liquid; D–Dry powder; N–Nose; NL–Nose and Lung.

Group	Immunization	Presentation	Route	Intended Deposition	Immunogenicity	Protective Efficacy
1	Vaccine	Spray-dried (reconstituted)	Intramuscular	Muscle	Lot: V-L	Lot: V-L
					N = 16	N = 10
2	Adjuvant-only	Spray-dried (reconstituted)	Intranasal	Nose	Lot: A-L	Lot: A-L
					N = 16	N = 10
3	Vaccine	Spray-dried (reconstituted)	Intranasal	Nose	Lot: V-L	Lot: V-L
					N = 16	N = 10
4	Adjuvant-only	Spray-dried - large particle	Dry aerosol	Nose	Lot: A-D-N	Lot: A-D-N
					N = 8 (×2)	N = 10
5	Vaccine	Spray-dried - large particle	Dry aerosol	Nose	Lot: V-D-N	Lot: V-D-N
					N = 8 (×2)	N = 10
6	Adjuvant-only	Spray-dried - small particle	Dry aerosol	Nose + Lungs	Lot: A-D-NL/1	Lot: A-D-NL/2
					N = 8 (×2)	N = 10
7	Vaccine	Spray-dried - small particle	Dry aerosol	Nose + Lungs	Lot: V-D-NL	Lot: V-D-NL
					N = 8 (×2)	N = 10

Each of the routes targeting respiratory delivery was also tested with adjuvant-only formulations that did not contain the antigen as negative controls (Group 2, 4, 6). Immunogenicity studies and protective efficacy studies were completed for each experimental group. Immunogenicity studies were conducted on 16 mice per group and protective efficacy studies were conducted on 10 mice per group. The maximum number of mice that could fit within the aerosol delivery device at a time was 12; therefore, the immunogenicity studies on the inhaled dry powder Groups 4, 5, 6, and 7 were completed in two sets of 8. The animal experiments in Table 1 were repeated once, although some of the specific immunogenicity readouts were not repeated in order to accommodate a larger diversity of complementary readouts between studies.

### Formulation Development of Suitable Inhalable Particles for Mice

Two formulations were designed in order to assess both intranasal and pulmonary delivery via dry powder inhalation. The aerodynamic diameter, $d_{\text{a}}$ , of an aerosol can be used as an important predictor of deposition site in respiratory systems. Kuehl et al. (Kuehl et al., 2012) conducted a study on the deposition of polydisperse aerosols in rodents as a function of aerosol $\;d_{\text{a}}$ . Of the particle sizes investigated in the study, they found that a minimum $d_{\text{a}}$ of 5 µm was required for nose-only deposition for mice (Kuehl et al., 2012). A maximum $d_{\text{a}}$ of 3 µm was required to obtain at least some lung deposition for mice, with lung deposition increasing with decreasing $d_{\text{a}}$ (Kuehl et al., 2012). Based on these findings, the formulation to assess intranasal delivery through nose-only deposition was designed to have a $d_{\text{a}}$ ≈ 5 μm, and the formulation to assess pulmonary delivery through both nose and lung deposition was designed to have a $d_{\text{a}}$ of 1–2 µm.

Assuming as a first approximation that the spray-dried particles have no voids and are spherical, their geometric diameter, $\;d_{\text{g}}$ , can be estimated using Eq.1, where $c_{\text{F}}$ is the solids concentration of the feedstock, $\rho_{\text{t}}$ is the true density of the particle, and $d_{\text{D}}\;$ is the diameter of the atomized droplets. Based on this equation, it is apparent that the size of particles can be influenced by modifying the feedstock concentration or by adjusting the processing conditions to change the atomized droplet diameter.

$$d_{\text{g}}=\sqrt[{3}]{\frac{c_{\text{F}}}{\rho_{\text{t}}}}d_{\text{D}}$$ (1)

The true density of the particles can be calculated using Eq.2, where $Y_{i}$ is the mass fraction of a given component and $\rho_{i}$ is the component’s true density. The density of the spray-dried vaccines can be approximated using the densities of the excipients trehalose, 1,580 kg/m^3^ (Grasmeijer et al., 2016), and trileucine, 1,250 kg/m^3^ (Carrigy et al., 2020). These values were used to calculate appropriate spray drying conditions suitable for manufacturing the powder at the targeted particle sizes.

$$\rho_{\text{t}}=\frac{1}{\sum_{i}\frac{Y_{i}}{\rho_{\text{t},i}}}$$ (2)

### Materials and Formulation Composition

The feedstock was prepared similarly to previous studies on spray drying the ID93+GLA-SE vaccine (Gomez et al., 2021a; Gomez et al., 2021b; Gomez et al., 2021c). All solutions were prepared with deionized water. Trehalose dihydrate with a purity of 98% (CAS 6138-23-4; Fisher Scientific Ottawa, ON, Canada) was used as the primary stabilizing agent. Formulations designed for dry powder delivery included trileucine with a purity of ≥90% (CAS 10329-75-6; Sigma Aldrich, Oakville, ON, Canada) as a dispersibility enhancing agent. All formulations included a buffer system consisting of Tris (hydroxymethyl) aminomethane (Tris) (CAS 77-86-1; Sigma Aldrich, Oakville, ON, Canada) and hydrochloric acid (CAS 7647-01-0; Sigma Aldrich, Oakville, ON, Canada) adjusted to a pH of 7.5.

The ID93 antigen and GLA-SE adjuvant components of the vaccine were produced separately. The construction, expression, and purification of the ID93 recombinant fusion protein has been described previously (Bertholet et al., 2010). Briefly, ID93 was expressed in *E. coli*, purified under denaturing conditions by chromatography, and analyzed by SDS-PAGE. GLA-SE was formulated with squalene droplets and dimyristoyl-sn-glycero-3-phosphocholine (DMPC) as an emulsifier. Manufacture of GLA-SE generally followed the same procedure as described in Orr et al. (Orr et al., 2014), except that the oil phase in the present work included the addition of α-tocopherol (0.05% w/v) and that glycerol and buffer were omitted from the aqueous phase. Three different lots of varying GLA concentrations were manufactured to achieve the target delivered GLA dose. All lots had an initial emulsion droplet size of 91–107 nm in diameter and low polydispersity indexes. ID93 protein was stored at a concentration of 1.2 mg/ml at −80°C prior to use. GLA-SE nanoemulsions with a squalene concentration of 10% v/v and GLA concentrations of 50 μg/ml or 5 mg/ml were stored in a refrigerator prior to use. All formulation processes began with preparation of 4 mg/ml Tris. The Tris solution was then pH adjusted by addition of hydrochloric acid to a pH of 7.5 ± 0.1. For each formulation, the trehalose and trileucine (if added) were dissolved in water into buffered Tris solution. Once fully dissolved, GLA-SE was added to the solution and gently mixed. ID93 was added last to the feedstock to minimize potential protein binding to container surfaces.

The custom aerosol delivery system was tested for feasibility and optimized prior to conducting the mouse study. This system was characterized with two vehicle (vaccine-free) formulations that were designed to be representative of the formulations used in the mouse study. The first formulation, C1, was designed to represent the powders intended for deposition in the nose following aerosolized dry powder delivery. The second formulation, C2, was designed to represent the powders intended for deposition in the nose and lungs after dry powder aerosolization. These representative powders were designed to have the same feedstock concentration of the trehalose, trileucine, and Tris components as their murine study counterparts. The feedstock concentrations and powder compositions of the vehicle-only spray-dried powders were as follows: C1 consisted of trehalose, trileucine, and Tris buffer with feedstock concentrations of 100 mg/ml, 3.3 mg/ml, and 2.4 mg/ml, respectively. C2 consisted of trehalose, trileucine, and Tris buffer with feedstock concentrations were 3.3 mg/ml, 0.11 mg/ml, and 0.08 mg/ml, respectively. The corresponding trehalose, trileucine, and Tris buffer mass fractions in both powders were 94.6, 3.1, and 2.3%, respectively. These spray-dried powders were used to optimize the aerosol delivery system while eliminating the chance of aerosolized exposure to ID93+GLA-SE.

The manufactured lots used for each murine study experiment were listed previously in Table 1. For consistency, one batch of powder was manufactured per experimental group, including replicates. However, the V-L and A-D-NL powders had to be completed in two batches due to limitations in processing capability. The V-L powder consisted of two separately prepared batches (V-L/1, and V-L/2) that were mixed in a 1:1 ratio by mass. This was not done for the A-D-NL/1 and A-D-NL/2 powders due to inability to guarantee evenly dispersed powders for aerosolization. This was not a concern for the V-L powder as it was intended for reconstitution prior to administration. Characterization of the powders was performed for each batch.

The solution preparation and mass fraction of the resulting powder for each formulation are given in Table 2 and Table 3, respectively. The formulations designed for reconstitution prior to delivery were based on a spray-dried ID93+GLA-SE vaccine developed in a previous study (Gomez et al., 2021a). Similarly, formulations designed for dry powder aerosol delivery to the nose or nose and lungs were modified from a human inhalable spray-dried presentation of ID93+GLA-SE investigated in a previous study (Gomez et al., 2021c).

**TABLE 2:** Solution preparation for manufacture of each experimental group composition. Nomenclature: V–Vaccine; A–Adjuvant; L–Liquid; D–Dry powder; N–Nose; NL–Nose and Lung.

Component	Group 1 and 3	Group 2	Group 4	Group 5	Group 6	Group 7
	(V-L)	(A-L)	(A-D-N)	(V-D-N)	(A-D-NL)	(V-D-NL)
Trehalose	100 mg/ml	100 mg/ml	100 mg/ml	100 mg/ml	3.33 mg/ml	3.33 mg/ml
Trileucine	—	—	3.9 mg/ml	3.9 mg/ml	0.128 mg/ml	0.128 mg/ml
Tris (buffer)	2.4 mg/ml	2.4 mg/ml	2.4 mg/ml	2.4 mg/ml	0.081 mg/ml	0.081 mg/ml
Squalene	17.2 mg/ml	17.2 mg/ml	17.2 mg/ml	17.2 mg/ml	0.57 mg/ml	0.57 mg/ml
DMPC	3.8 mg/ml	3.8 mg/ml	3.8 mg/ml	3.8 mg/ml	0.13 mg/ml	0.13 mg/ml
GLA	0.01 mg/ml	0.01 mg/ml	1 mg/ml	1 mg/ml	0.033 mg/ml	0.033 mg/ml
ID93	0.004 mg/ml	—	—	0.4 mg/ml	—	0.027 mg/ml

**TABLE 3:** Mass fraction composition of each formulation. Nomenclature: V–Vaccine; A–Adjuvant; L–Liquid; D–Dry powder; N–Nose; NL–Nose and Lung. *Expected ID93 mass fraction for Group 7 calculated from solution preparation assuming ∼50% processing loss.

Component	Group 1 and 3	Group 2	Group 4	Group 5	Group 6	Group 7
	(V-L)	(A-L)	(A-D-N)	(V-D-N)	(A-D-NL)	(V-D-NL)
**Trehalose**	81.0%	81.0%	77.9%	77.9%	77.9%	77.9%
**Trileucine**	—	—	1.9%	1.9%	1.9%	1.9%
**Tris (buffer)**	2.0%	2.0%	3.0%	3.0%	3.0%	3.0%
**Squalene**	13.9%	13.9%	13.4%	13.4%	13.4%	13.4%
**DMPC**	3.1%	3.1%	3.0%	3.0%	3.0%	3.0%
**GLA**	0.008%	0.008%	0.8%	0.8%	0.8%	0.8%
**ID93**	0.003%	—	—	0.3%	—	0.3%*

Optimization experiments with the C1 and C2 formulations (see Results) demonstrated that it would not be possible to deliver the target dose of 0.4 µg of ID93 and 1 µg of GLA through dry powder inhalation with the same mass fraction of ID93 and GLA as the powders intended for reconstitution. The main constraint was the tolerable aerosol concentration and dosing time for the mice. To achieve the target delivered dose, the ID93 and GLA concentrations in the dry powder aerosol formulations were concentrated 100×. However, it was not possible to similarly concentrate squalene and emulsifier content due to practical limitations. Therefore, whereas ID93 and GLA dose were intended to be constant regardless of formulation phase or route of delivery, squalene dose was ∼100-fold lower in the dry powder aerosol formulations for nasal or pulmonary delivery compared to the reconstituted liquid formulations. Nevertheless, the impact of this limitation was expected to be minimal since our previous work indicated that squalene was not necessary for immunogenicity or protective efficacy following intranasal immunization with ID93 + GLA (Orr et al., 2015). Finally, previous development work (data not shown) suggested that manufacturing of the powders for nose and lung delivery resulted in greater ID93 processing loss due to the harsher processing conditions needed to achieve the required small particle size. Therefore, the solution concentration of ID93 in the V-D-NL formulation was increased further relative to the other formulations, assuming an approximately 50% processing loss.

### Spray Drying

Spray drying was conducted using similar equipment to that described in previous studies (Gomez et al., 2021a; Gomez et al., 2021b; Gomez et al., 2021c). Briefly, the feedstock was supplied to an atomizer using a peristaltic pump. The feedstock was subsequently atomized into a custom-built research spray dryer. The powders were collected into glass jars at the outlet of the spray dryer. The collected powders were stored in a dry environment prior to packaging.

The processing parameters used for spray drying the C1 and C2 formulations for the characterization of the RBG-NOID were as follows: C1 was processed with a drying gas flow rate of 500 SLPM, an inlet temperature of 70°C, an atomizing gas pressure of 69 kPa, a liquid feed flow rate of 1.8 ml/min, a predicted outlet temperature of 46°C, a predicted outlet RH of 6%, and an atomizer air-liquid ratio of 3; C2 was processed with a drying gas flow rate of 800 SLPM, an inlet temperature of 70°C, an atomizing gas pressure of 552 kPa, a liquid feed flow rate of 3.3 ml/min, a predicted outlet temperature of 49°C, a predicted outlet RH of 8%, and an atomizer air-liquid ratio of 10. These spray drying conditions were calculated iteratively using an energy and mass balance model (Ivey and Vehring, 2010) to maximize or minimize the initial atomized droplet size for the C1 and C2 powders, respectively. A relatively low outlet temperature and relative humidity was also prioritized to reduce losses at the collection point. Based on the processing conditions and feedstock concentration, the theoretical mass median diameter was 4.6 and 1.1 µm for the C1 and C2 formulations, respectively.

The processing parameters used to spray dry the formulations for the mouse studies are given in Table 4. The conditions used to spray dry the formulations designed for reconstitution prior to delivery (V-L and A-L) were the same as used in a previous study (Gomez et al., 2021a). The conditions used to spray dry the powders for dry powder administration to the nose (A-D-N and V-D-N) were similar to those used to spray dry the C1 formulation. The conditions used to spray dry the powders for aerosolized dry powder administration to the nose and lungs (A-D-NL and V-D-NL) were similar to those used to spray dry the C2 formulation. Both of these conditions were chosen to maximize or minimize the initial droplet diameter to achieve larger or smaller particles, respectively. Collected powders were packaged to minimize moisture exposure in the time between manufacture and usage. This packaging method was used successfully in previous stability studies (Gomez et al., 2021a; Gomez et al., 2021c). Briefly, powders were aliquoted into separate low-bind snap cap tubes by mass based on the given experiment. Powder-containing tubes were individually packaged in aluminum bags along with a desiccant pouch and heat sealed. These packages were then placed within another aluminum bag with a desiccant pouch and heat sealed. The packages were kept refrigerated prior to use.

**TABLE 4:** Spray drying processing parameters used for manufacturing powder for preclinical trials. Nomenclature: V–Vaccine; A–Adjuvant; L–Liquid; D–Dry powder; N–Nose; NL–Nose and Lung.

Parameters	Group 1/2/3 (V-L and A-L)	Group 4/5 (A-D-N and V-D-N)	Group 6/7 (A-D-NL and V-D-NL)
**Drying Gas Flow Rate (SLPM)**	200	400	750
**Inlet Temperature (°C)**	65	70	75
**Atomizing Gas Pressure (kPa)**	69	69	552
**Liquid Feed Flow Rate (ml/min)**	0.6	1.8	3.3
**Predicted Outlet Temperature (°C)**	36	43	50
**Predicted Outlet RH (%)**	7	8	8

### Characterization of Aerosol Delivery System

**FIGURE 1:**
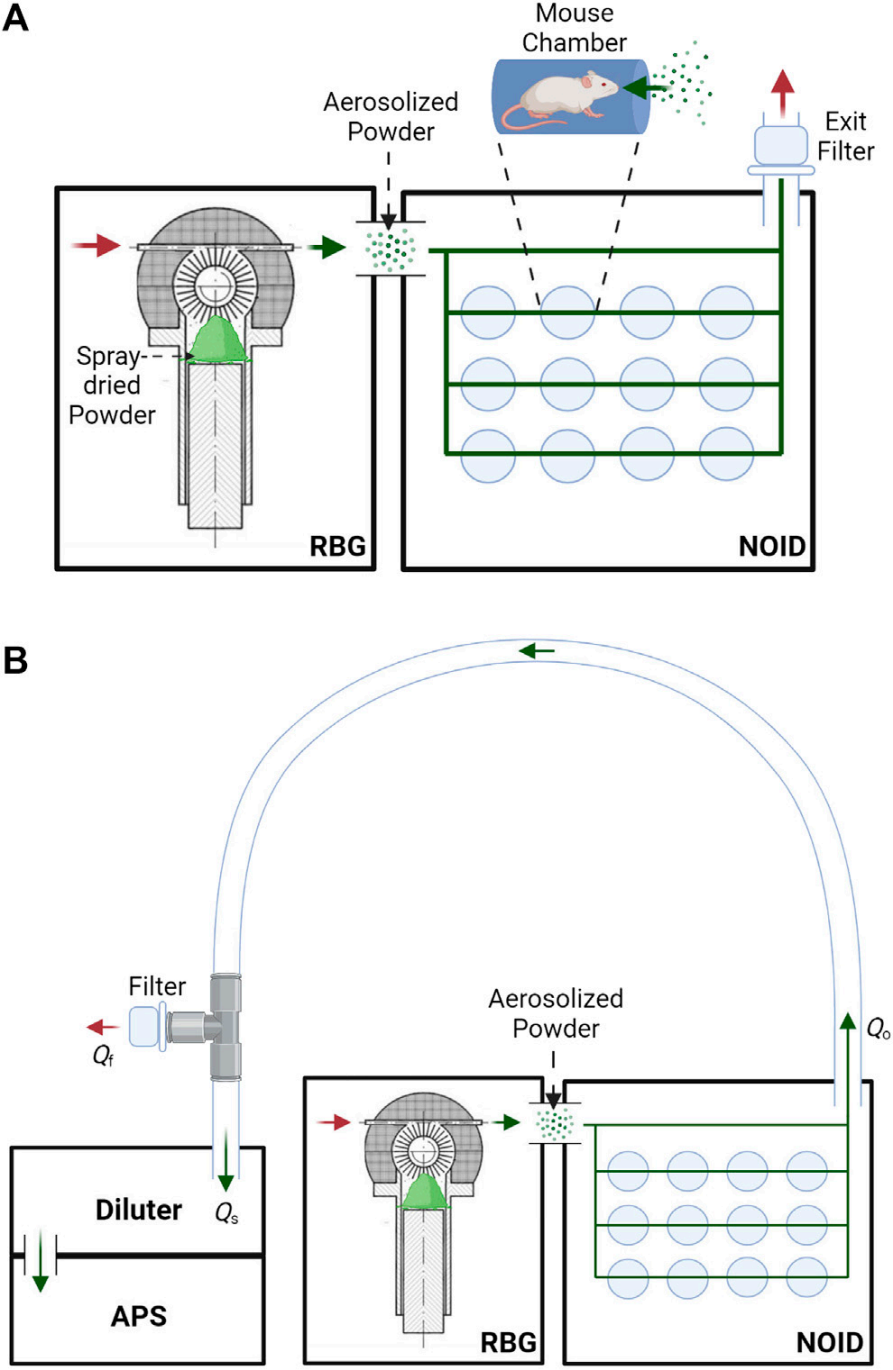
**(A)**: Schematic of the aerosol delivery system, consisting of the RBG system (Phillips et al., 2017) connected to the NOID. Spray-dried powder is loaded into the RBG system, which then aerosolizes the powder. The powder aerosol traverses the NOID to be inhaled through the nose of the mice. Red arrows represent the flow of clean air, and green arrows represent the flow of aerosolized powder. **(B)**: Simplified schematic of the system used to assess aerosol particle size at the outlet of the aerosol delivery system. Aerosol particle size at the outlet of the RBG-NOID was measured using an aerodynamic particle sizer (APS). Abbreviations and nomenclature: RBG–Rotating Brush Generator, NOID–Nose-Only Inhalation Device, APS–Aerodynamic Particle Sizer, *Q*_o_–flow rate at the outlet of the RBG-NOID apparatus (8.33 L/min), *Q*_s_–sampling flow rate of the APS (5 L/min), *Q*_f_–flow rate of the air exiting the filter from the tee fitting. Figures created with BioRender.com.

This study used a nose-only exposure system to dose the mice with the dry powder, instead of the insufflation method that was often used in the past. Although insufflation is efficient, this method does not allow for realistic aerosol particle sizes and deposition patterns and is thus not representative of inhalation in humans. A rotating brush generator (RBG) system (RBG 1000G; Palas GmbH, Karlsruhe, Germany) was utilized to disperse the spray-dried powder into an aerosol. The configuration used for this study included a 7 mm diameter feedstock reservoir and dispersion cover type C. The RBG device was connected to a modified version of a custom-made nose-only inhalation device (NOID), first developed by Nadithe et al. (Nadithe et al., 2003). The modified version of the NOID has been used before in immunogenicity and protective efficacy mouse studies with nebulized bacteriophage (Carrigy et al., 2019). A simplified schematic of the RBG and NOID system (RBG-NOID) with an exit filter is shown in Figure 1 (top).

The spray-dried powder and aerosol delivery system was designed for preclinical trials involving C56BL/6 mice. The approximate average respiratory minute volume, $V_{m}$ , was calculated to be 22 ml/min per mouse for the same strain of mice (Carrigy et al., 2019). This value is similar to the generally given $V_{m}$ for laboratory mice (23 ml/min) (Cryan et al., 2007). The maximum respiratory minute volume is 264 ml/min given that the maximum number of mice that can be housed in the NOID is 12. Including a safety factor of 2, the minimum aerosol flow rate to prevent a hypoxic environment for the mice is 528 ml/min. All experiments were conducted at the lowest possible system air flow rate, 0.5 m^3^/h (8.33 L/min). The lowest flow rate was chosen to minimize the difference between the mouse inhalation rate and the aerosol flow rate and thus increase the aerosol available to the mice. All experiments were conducted at the maximum brush-rotating speed, 1,200 rpm, to maximize the dispersing force.

Powder was loaded into the RBG reservoir within a dry environment in order to minimize moisture uptake. The mass of the powder loaded into the reservoir (nominal dose) was recorded as the difference in the powder stock container mass before and after loading powder. Mouse noseports were plugged for the characterization experiments. Experiments consisted of aerosolizing the C1 or C2 powder using the RBG and determining the delivery efficiency through the NOID. Two piston feed rates were assessed: ∼300 mm/h and 150 mm/h. Repeat experiments at ∼300 mm/h were conducted to assess repeatability of the results. An exit filter (VP7100; KEGO Corporation, London, ON, Canada) was placed at the outlet of the NOID and was measured gravimetrically before and after each experiment to determine powder deposition on the exit filter. Airflow was run for an additional minute after all powder was aerosolized by the RBG in order to allow any remaining aerosol to traverse the system. The equipment was completely cleaned and dried between each experiment.

Lower piston feed rates were not investigated due to a selected duration of exposure limit of 20 minutes. Mice have been reported to tolerate restraint tubes for less than an hour, even after acclimation to the devices (Phillips et al., 2017). Retention of the mice in the tubes for longer periods of time may lead to stress-induced breathing pattern changes. Additionally, a maximum of 3 mg/L of aerosol concentration at the noseports was set to limit mouse distress due to high aerosol concentration. The aerosol concentration at the noseports, $C_{\text{n}}$ , can be calculated using Eq.3, where $m_{\text{n}}$ refers to the mass of spray-dried powder delivered to the noseports and D is the duration of exposure.

$$C_{\text{n}}=\frac{m_{\text{n}}}{V_{m}\times D}$$ (3)

The mass of powder that deposited on the exit filter was used to estimate the amount of powder that was delivered to each of the noseports using a ratio of flow rates, as given in Eq.4. In this equation, $m_{\text{f}}$ refers to the measured mass of powder deposited on the exit filter, and $Q_{\text{o}}$ is the aerosol flow rate at the outlet of the RBG-NOID device (8.33 L/min). The delivered dose is defined as the dose available for breathing, as compared to the deposited dose, which is defined as the dose that deposits in the lung. This distinction was made explicit as the deposited dose is expected to be much lower than the delivered dose, with the FDA estimating that only 10% of aerosol delivered to rodents will reach the respiratory system (Tepper et al., 2016).

$$\frac{m_{\text{n}}}{m_{\text{f}}}=\frac{V_{m}}{Q_{o}}$$ (4)

Following the optimization characterization experiments, a feed rate of 150 mm/h was chosen to assess the dispersing capabilities of both the spray-dried C1 and C2 formulations. The particle size distribution at the outlet of the aerosol delivery system was measured to determine if the tested powder was adequately dispersed. A flexible hose was connected at one end to the outlet of the NOID and connected at the other end to an aerosol diluter (Aerosol Diluter 3302A; TSI, Shoreview, MN, United States) mounted on a time-of-flight aerodynamic particle sizer (APS) (Aerodynamic Particle Sizer Spectrometer 3,321; TSI, Shoreview, MN, United States). The aerosol flow rate of the RBG-NOID at operation was 8.33 L/min and the sampling flow rate of the APS was 5 L/min. To prevent pressure buildup within the system, a tee fitting with a filter (VP7100; KEGO Corporation, London, ON, Canada) was attached to the hose prior to the diluter and APS system. A simplified schematic of this sampling setup is shown in Figure 1. Clear, flexible tubing was used to transfer the aerosol from the outlet of the RBG-NOID to the sampling system. Flexible tubing was used to avoid sharp changes in flow direction as gently curved streamlines will mitigate large particle deposition as compared to abrupt changes during aerosol transportation through the tubing.

The APS device measures the count median aerodynamic diameter, *CMAD,* and the geometric standard deviation, $\sigma_{g}$ . The mass median aerodynamic diameter, *MMAD*, was calculated using the Hatch-Choate equation, as shown in Eq.5. Six and nine recordings were obtained for the C1 and C2 formulations, respectively.

$$MMAD=CMAD\cdot e^{3\ln^{2}\left(\sigma_{g}\right)}$$ (5)

### Characterization of Spray-Dried Formulations

#### Scanning Electron Microscopy

Assessment of particle morphology was completed using Field Emission Scanning Electron Microscopy (Zeiss Sigma FE-SEM; Carl Zeiss, Oberkochen, Germany). Powder was mounted onto a carbon tape-covered aluminum SEM stub (Product 16,111; Ted Pella, Inc.; Redding, CA, United States), and subsequently samples were sputtered with a gold coating (Denton Vacuum Desk II Sputter Coater; Denton, Moorestown, NJ, United States) to a thickness of approximately 16 nm. Images were taken at a magnification of 3,000–5,000x.

#### Dynamic Light Scattering

For each formulation, the size of the GLA-SE nanoemulusion droplets was assessed after reconstituting the spray-dried formulation back to the feedstock concentration. Mean hydrodynamic diameter and polydispersity of the nanoemulsion droplets were measured using dynamic light scattering with a measurement angle of 173° (NanoZS; Malvern, Worcestershire, United Kingdom). The mean hydrodynamic diameter and polydispersity index were calculated by the instrument software from a cumulants analysis of the intensity autocorrelation function.

#### Reverse-phase HPLC

Squalene and GLA content for each formulation were quantified after reconstitution by reversed phase HPLC using an Agilent 1200 HPLC (Agilent Technologies; Santa Clara, CA, United States) equipped with a silica-based, C18 reversed-phase column (Atlantis T3 Column; Waters; Elstree, United Kingdom) held constant at 30°C. Each analyte was detected using a charged aerosol detector (Corona CAD; ESA Biosciences; Chelmsford, MA, United States). Mobile phase A contained 75:15:10 (v/v/v) methanol:chloroform:water, 1% (v/v) acetic acid, and 20 mM ammonium acetate, and mobile phase B contained 50:50 (v/v) methanol:chloroform, 1% (v/v) acetic acid, and 20 mM ammonium acetate. Samples were diluted in mobile phase B and injected with a gradient over 30 min for squalene content analysis or 18 min for GLA content analysis. Squalene content was quantified by peak area and GLA content was quantified by peak height. Concentration measurements were made by interpolation from a curve generated from standards fitted with a second order polynomial. Samples were diluted in mobile phase B at different factors depending on the expected GLA and squalene content. Groups 1–3 were diluted 1:10 for GLA and 1:100 for squalene. Groups 4-5 were diluted 1:1,250 (1:5 and 1:10 with water, 1:25 with mobile phase B in serial) for GLA and 1:100 for squalene. Groups 6–7 were diluted 1:25 for GLA and 1:10 for squalene.

#### SDS-PAGE

ID93 concentration was quantified after reconstitution back to the feedstock concentration using densitometry analysis of reducing SDS-PAGE based on a standard curve. Samples were prepared by mixing a 20% (w/v) sodium dodecyl sulfate solution (Thermo Fisher Scientific, Waltham MA, United States), 4X LDS Buffer (Thermo Fisher Scientific, Waltham, MA, United States) spiked with 5% (v/v) β-mercaptoethanol, and reconstituted sample in a 2:1:1 ratio. Due to the elevated ID93 concentration, prepared Group 5 (V-D-N) samples were diluted 1:50 to bring ID93 concentration into the range of the standard curve. The upper limit of quantitation of the assay is 0.02 mg/ml ID93. Samples were heated for 15 min at 85°C and loaded into a 4–20% Tris-Glycine SDS-PAGE gel (Thermo Fisher Scientific, Waltham, MA, United States). The gel was run at 180V for 65 min and then stained overnight using a SYPRO Ruby stain (Thermo Fisher Scientific, Waltham, MA, United States) and imaged (ChemiDoc; Bio-Rad, Mississauga, ON, Canada). ID93+GLA-SE standards at 10 ng, 50 ng, and 100 ng protein load were prepared in the same manner and included on each gel. Densitometry analysis was performed using Image Lab 6.0 software (Bio-Rad Laboratories, Hercules, CA, United States). The three standards were used to generate a standard curve based on band intensity and ID93 was interpolated from the standard curve.

### Mice, Immunizations, Aerosol Challenge, and Sample Collection

C57BL/6J (B6) (Jackson Laboratories, Bar Harbor, ME, United States) mice were bred under specific pathogen-free conditions at the Infectious Disease Research Institute (IDRI) (for the initial experiment shown in Supplementary Figure S1) or at the Washington University in St. Louis (for all other animal studies)*.* Mice were used at 6–8 weeks of age. All animal experiments were performed in accordance with National and Institutional guidelines for animal care of laboratory animals and approved by the Washington University in St Louis Institutional Animal Care and Use Committee (IACUC) under protocol 20190101 or by the IDRI IACUC under protocol 2019-6.

For the initial study (Supplementary Figure S1), lyophilized or spray-dried batches of ID93-GLA-SE were reconstituted prior to immunization. Cohorts of 5 female mice per group were immunized once via intramuscular injection in the calf muscles of hind limbs with 100 μl (50 µl/leg) of either the vehicle only (10% trehalose + 20 mM Tris pH 7.5 in water) or 0.4 μg of ID93 and 1 μg of GLA in a 2% stable squalene oil emulsion (SE). Spleens and draining inguinal lymph nodes were collected in RPMI 7 days post immunization. Cell suspensions were obtained by manual disruption. Red blood cells contained in spleens were lysed using the Red Blood Cell Lysis Buffer (eBioscience, San Diego, CA, United States). Central blood was collected by cardiac puncture from mice under deep anesthesia on day 7. Serum was separated from whole blood by centrifugation at 10,000 rpm for 5 min and was stored at -70°C until use.

For all subsequent studies, equal numbers of male and female mice were immunized with ID93+GLA-SE or only GLA-SE through intramuscular (IM) liquid injection, intranasal (IN) liquid delivery, or dry powder aerosol delivery to the nose or nose and lungs, as described in Table 1, at day 0 and at day 21. Mice were anesthetized using ketamine (80 mg/kg) (AKorn Animal Health Inc., Lake Forest, IL, United States) and xylazine (6 mg/kg) (AKorn Animal Health Inc., Lake Forest, IL, United States) to restrain the mice inside the NOID device for the aerosol-inhalation immunizations. Whereas for IN immunizations, mice were anesthetized using isoflurane (Henry Schein Animal Health, Dublin, OH, United States). In the immunogenicity studies, 5 mice (out of 224 total mice) died 24–48 h following immunizations (1 mouse administered liquid nasal GLA-SE, 1 mouse administered nasal aerosol GLA-SE, and 3 mice administered nasal aerosol ID93+GLA-SE). In the protective efficacy studies, 5 mice (out of 140 total mice) died following immunizations (1 mouse administered liquid nasal GLA-SE, 1 mouse administered nasal aerosol ID93+GLA-SE, 2 mice administered pulmonary aerosol GLA-SE, and 1 mouse administered pulmonary aerosol ID93+GLA-SE). In the protective efficacy studies, mice were challenged 4 weeks after the second immunization by aerosol with a low dose [100 colony forming units (CFU)] of *Mtb* strain H37Rv (BEI Resources, Manassas, VA, United States) using a Glas-Col airborne infection system (Glas-Col LLC, Terre Haute, IN, United States). Four weeks after challenge, unvaccinated and vaccinated mice were sacrificed by carbon dioxide (CO_2_) asphyxiation, and the lungs were aseptically excised and individually homogenized in physiological saline solution. Serial dilutions of lung and spleen homogenates were plated on 7H11 selective agar (BD bioscience, San Diego, CA, United States) for *Mtb* CFU and counted after 3 weeks of incubation at 37°C as described before (Nakae et al., 2002).

For the immunogenicity experiments, lung and spleen single-cell suspensions from immunized, unchallenged mice were isolated after the second immunization as previously described (Ardain et al., 2019). Briefly, mice were euthanized with CO_2_ and lungs were perfused with heparin in saline. Harvested lungs were minced and incubated in collagenase/DNAse for 30 min at 37°C. Lung and spleen tissues were pushed through 70 µm nylon screens to obtain single-cell suspension. Red blood cells were lysed with Gey’s Balanced Salt Solution (Sigma-Aldrich, St. Louis, MO, United States), and the cells were resuspended in complete DMEM (DMEM+10% FBS) for downstream analysis such as flow cytometry and ELISA assays. Bronchoalveolar lavage (BAL) was isolated from immunized animals as previously described (Gopal et al., 2013; Slight et al., 2013). Briefly, the chest cavity was opened and the sternum/ribcage was resected. The trachea was isolated and a blunt tipped needle was gently inserted into the trachea. The lungs were lavaged with 1 (1 × 1 ml) wash with sterile 0.2 mM EDTA (Sigma-Aldrich, St. Louis, MO, United States) in PBS. Bone marrow was harvested and processed as previously described (Griffiths et al., 2016a) at 1 week and 4 weeks after the second immunization. Briefly, cells were isolated from the femur and tibia of the immunized animals. Red blood cells were lysed with Gey’s Balanced Salt Solution, and the cells were resuspended in cDMEM for ELISpot and antibody ELISA assay.

#### Flow Cytometry Staining

In the initial mouse immunogenicity experiment (Supplementary Figure S1), cells were incubated with the I-A(b) *Mtb* Rv3619 63–73 VIYEQANAHGQ tetramer (NIH Tetramer Core Facility at Emory University, Atlanta, GA, United States) and Fc receptor block (anti-CD16/32 antibody, eBioscience) for 1 h at 37°C. Cells were then surface stained with CXCR5, CD8, B220, CD11b, PD-1, CD4, and CD44. Cells were subsequently permeabilized in Foxp3/Transcription factor Fix and Perm buffer (eBioscience) for 1 h at RT and then stained overnight at 4°C with FoxP3 and T-bet. Cells were gated as singlets > lymphocytes > CD4^+^ CD8^−^ B220- CD11b- > Tetramer + CD44^+^ > CXCR5+ PD-1+ (TFH) or CXCR5- PD1- FoxP3- T-bet+ (TH1) or CXCR5- PD1- FoxP3 (Treg). A second panel for intracellular cytokine staining was performed where cells were stimulated for 2 h with media (RPMI 1640 + 10% FCS) or ID93 (10 μg/ml) at 37°C and subsequently incubated with Brefeldin A (eBioscience) for an additional 8 h at 37°C. Cells were surface stained with CD4, CD8, B220, CD11b, and CD44 together with Fc receptor block, followed by permeabilization with Cytofix/Cytoperm (BD Biosciences) and intracellular staining with CD154, TNF, IL-2, GM-CSF, IL-17A, IL-5, and IFN-γ. Cells were gated as singlets > lymphocytes > CD4^+^ CD8^−^ B220- CD11b- > CD44^+^ > cytokine+. The complete antibody panels, including fluorochrome, dilution factor, and manufacturer are listed in Supplementary Table S1, and representative gating strategies are represented in Supplementary Figure S2.

In the subsequent mouse immunogenicity experiment, the antibodies CD154, TNF, CD44, IL-5, IFN-γ, GMCSF, CD8, IL-17A, CD4, and IL-2 were employed. The complete antibody panels, including fluorochrome, dilution factor, and manufacturer are listed in Supplementary Table S1. Cells were stimulated with ID93 protein (10 μg/ml), along with Brefeldin A (BioLegend). Following the antigen stimulation, the cells were stained in 96-well U-bottom plates using the LIVE/DEAD™ Fixable yellow Dead Cell Stain Kit (Thermo Fisher Scientific, Waltham, MA, United States) as per the manufacturer’s protocol. The cells were then stained for surface markers for 30 min. Intracellular cytokine staining was performed using the BD Cytofix/Cytoperm kit (BD Biosciences, San Diego, CA) following manufacturer’s instructions. Intracellular staining with anti-IFN-γ, IL-2, TNF-α, IL-5, GMCSF, CD154, and IL-17 was performed for 30 min. Cells single stained with each fluorochrome were used as controls for the compensation matrix in the flow cytometry (Ardain et al., 2019). Samples were acquired on a 4 laser BD LSRII or X20 Flow Cytometer, and the analysis was performed using FlowJo software version 7.6.5 (Treestar, FlowJo, LLC, Ashland, OR, United States). The gating strategy is represented in Supplementary Figure S2.

#### Cytokine and Antibody Quantification Using Enzyme-Linked Immunosorbent Assay

For the initial mouse immunogenicity experiment, Corning high bind 384-well microtiter plates (VWR International, Radnor, PA, United States) were coated overnight at 4°C with 2 μg/ml ID93 in coating buffer (eBioscience). Plates were blocked for 2 h with 1% BSA-PBS, and 12-point 2-fold serial dilutions of the serum samples were carried out. Detection antibodies included anti-mouse IgG1, IgG2c, or total IgG conjugated to horse radish peroxidase (Southern Biotech, Birmingham, AL, United States). Plates were incubated with 3,3′,5,5′-Tetramethylbenzidine (TMB) for 5 min, and the reaction was stopped using 1 N H_2_SO_4_. Optical density (O.D.) readings were taken at 450 nm using an automated plate reader (ELx808 or Synergy 2, BioTek, Winooski, VT, United States).

For the subsequent mouse immunogenicity experiments, splenocytes (2 × 10^5^ cells/well) from immunized animals were stimulated with 2 μg/ml ID93 for 48 h. IL-17, IFN-γ and IL-5 were quantified in the supernatant by ELISA, according to manufacturer’s instructions (R and D Systems, Minneapolis, MN, United States). Total IgG, IgG1, IgG2a, and IgG2c were measured in the serum and BAL samples from the immunized animals using the reagents indicated below from Southern Biotech. Briefly, the 96-well plates were coated with 2 μg/ml ID93 antigen overnight at 4°C. The next day, plates were washed and blocked with 1% BSA. After washing, plates were incubated with the sera or BAL samples (diluted 5-fold and serially diluted up to 5 dilutions) followed by incubation at room temperature for 2 h with the HRP-conjugated antibodies against total IgG (Cat # 1,031-05), IgG1 (Cat # 1,070-05), IgG2a (Cat # 1,080-05) and IgG2c (Cat # 1,079-05), and TMB substrate (VWR, Radnor, PA, United States). The dilution for total IgG was 1:4,000, and for all other antibodies the dilution was 1:2000. The reaction was stopped using 1N H_2_SO_4,_ and the data were collected within 30 min using a plate reader (ELx405 BioTek, Winooski, VT, United States).

#### Antigen-specific Long-Lived Antibody-Secreting Plasma Cell Responses–B Cell ELISpot Assay

ELISpot plates (Millipore, Bedford, MA, United States) were coated with 2 μg/ml ID93 and incubated overnight at 4°C. Plates were washed with PBS, blocked with complete RPMI and 10% FBS for 2 h, at room temperature, and then washed again. Single-cell suspensions from harvested bone marrow were prepared as described above and seeded at 1.5×10^6^ cells per well. The plates were then incubated at 37°C with 5% CO2 for 3 h, washed, and HRP-conjugated anti-mouse IgG (H + L) or IgA antibodies (Southern Biotech, Birmingham, AL, United States) were added for overnight incubation at 4°C. The plates were developed with AEC substrate kits according to the manufacturer’s protocol (Vector Laboratories, Burlingame, CA, United States). Spots were counted using an automated ELISPOT reader (CTL. Analyzer, Cellular Technology Ltd., Shaker Heights, OH, United States). Data were analyzed using ImmunoSpot® software (CTL Analyzer).

#### Statistical Analysis

For *in vivo* efficacy and immunogenicity experiments, the differences between selected groups were analyzed using one-way or two-way ANOVA with appropriate correction for multiple comparisons as indicated using GraphPad Prism 9.2 (GraphPad Software; San Diego, CA, United States). In cases where standard deviations were significantly different according to the Brown-Forsythe test, Welch’s ANOVA test was employed with Dunnet’s T3 correction for multiple comparisons. The Kruskal-Wallis non-parametric test with Dunn’s correction for multiple comparisons was used when multiple comparisons were not possible with Welch’s ANOVA due to limitations of the latter test when one experimental group has constant values. A *p*-value of <0.05 was considered statistically significant.

## Results

### Optimization of Aerosol Delivery System and Formulation for Nasal and Pulmonary Delivery

**FIGURE 2:**
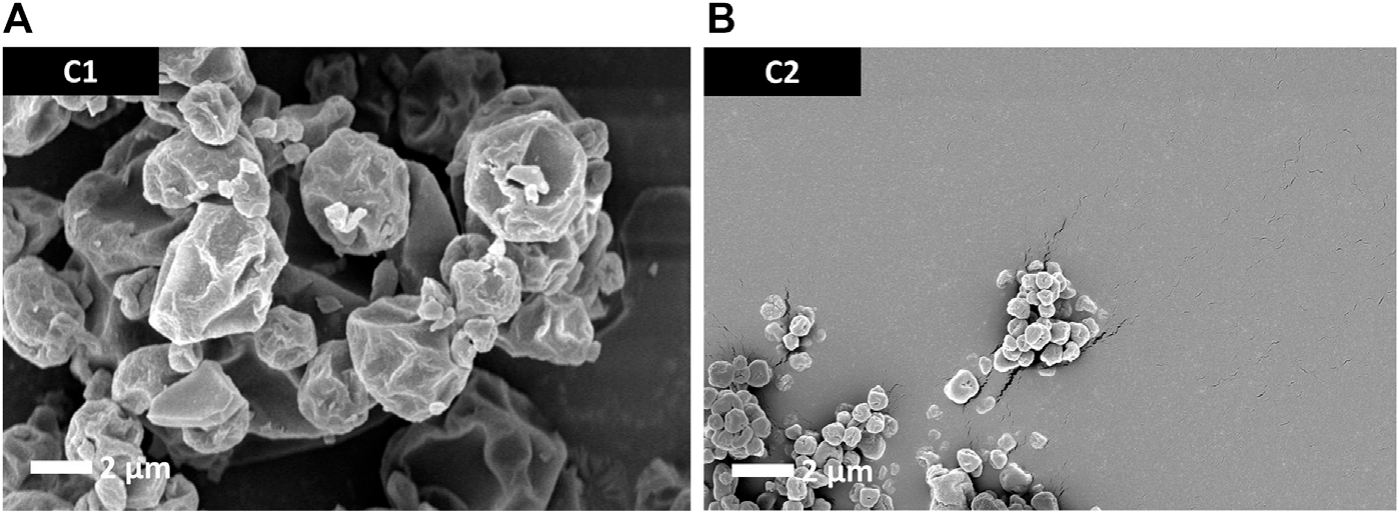
SEM images of the trileucine-containing C1 **(A)** and C2 **(B)** spray-dried powders. These vehicle powders were designed to not have the antigen or adjuvant system but still be representative of the spray-dried powders developed for the mouse study dry powder administration routes.

Particle morphology of the antigen-free trileucine-containing C1 and C2 formulations is shown in Figure 2. Both formulations showed rugose particle morphologies due to the inclusion of trileucine as a dispersibility enhancer. A rougher surface morphology enhances powder dispersibility and thus improves aerosol performance (Gomez et al., 2021b). The C1 formulation was significantly larger in particle size than the C2 formulation, as designed. Visually, both appeared to be close in size to their targeted value.

A summary of the optimization experiments for the spray-dried C1 formulation representing deposition in the nose of mice is given in Table 5. Tests 1 and 2 were completed at approximately the same feed rate in order to assess the dosing reproducibility; the delivered dose for these tests was 0.013 ± 0.005% nominal dose. Test 3 was completed at a lower feed piston rate and consequently demonstrated a higher efficiency of powder delivery compared to Test 1 and 2. All tests showed that the aerosol concentration at the noseports was less than 3 mg/L, and the duration of exposure was less than the 20 min limit. Based on these experiments, a feed rate of 150 mm/h or lower was recommended for maximizing the efficiency of dispersing the spray-dried vaccine powder targeting nasal deposition.

**TABLE 5:** Summary of RBG-NOID characterization experiments with the spray-dried C1 formulation. The C1 formulation was designed for deposition in the nose of mice upon inhalation. Abbreviations: RBG–rotating brush generator; NOID–nose only inhalation device.

Test ID	Piston Feed Rate (mm/hr)	Measured Values			Calculated Values		
		Nominal Dose (mg)	*D* (min)	*m*_f_ (% nominal dose)	*m*_n_ (% nominal dose)	*m*_n_ (mg)	*C*_n_ (mg/L)
1	303	690.1	6.7	3.29	0.009	0.06	0.41
2	304	711.0	7.3	6.10	0.016	0.12	0.71
3	150	495.9	11.4	8.93	0.024	0.12	0.47

A summary of the parameter optimization experiments for the spray-dried C2 formulation representing deposition in the nose and lungs of mice is given in Table 6. Tests 4, 5, and 6 were completed at approximately the same feed rate in order to assess the dosing reproducibility. For these tests, a delivered dose of 0.081 ± 0.009% nominal dose was achieved, indicating a similar level of reproducibility as the C1 formulation. These results showed that the C2 formulation had a higher delivery efficiency than the C1 formulation under the same operating conditions.

**TABLE 6:** Summary of RBG-NOID characterization experiments with the spray-dried C2 formulation. The C2 formulation was designed for deposition in the nose and lungs of mice upon inhalation. Abbreviations: RBG–rotating brush generator; NOID–nose only inhalation device.

Test ID	Piston Feed Rate (mm/hr)	Measured Values			Calculated Values		
		Nominal Dose (mg)	*D* (min)	*m*_f_ (% nominal dose)	*m*_n_ (% nominal dose)	*m*_n_ (mg)	*C*_n_ (mg/L)
4	306	852.1	8.0	33.4	0.088	0.75	4.25
5	306	754.1	9.2	27.0	0.071	0.54	2.65
6	304	754.7	6.6	32.0	0.084	0.64	4.39
7	150	637.2	13.7	40.0	0.11	0.67	2.23

The aerosol concentration at the noseports for experiments conducted at ∼300 mm/h feed rate was 3.76 ± 0.97 mg/L, above the set aerosol concentration limit. Reducing the feed rate increased the delivery efficiency and decreased the aerosol concentration at the noseports to tolerable levels. Based on these results, a feed rate of 150 mm/h or lower was recommended for maximizing the delivery efficiency while ensuring tolerable aerosol concentration at the noseports.

Based on the 0.003 and 0.008% mass fraction of ID93 and GLA, respectively, in the spray-dried powders developed for humans (Gomez et al., 2021a; Gomez et al., 2021c), approximately 13 mg of powder must be delivered to each mouse. The optimization experiments indicate that this powder dose could not be achieved under the tested conditions for either the C1 or C2 formulation. As explained previously, these experiments led to the decision to concentrate the ID93 and GLA components for the mouse study by 100× in the formulations produced for dry powder aerosol delivery to achieve the dosing target with a reduced amount of powder. However, it was not possible to similarly concentrate squalene and emulsifier content due to practical limitations. Therefore, squalene dose was ∼100-fold lower in the dry powder aerosol formulations for nose or nose and lung delivery compared to the reconstituted liquid formulations. However, previous work indicated that squalene was not necessary for protective efficacy following IN immunization with ID93 + GLA (Orr et al., 2015).

The C1 and C2 powders were aerosolized using the RBG-NOID system and sized to assess how well the aerosol system was dispersing the powders. The results of the size distribution measurements are shown in Table 7. As previously discussed, the $d_{\text{a}}$ targets were ∼5 μm and 1–2 µm for the formulations targeting nose-only deposition, and nose and lung deposition in mice, respectively.

**TABLE 7:** Measured particle size distribution of the dispersed C1 and C2 powders at the outlet of the RBG-NOID. Dispersion experiments were conducted at the optimized feed rate of 150 mm/h. Results shown are the average ± standard deviation of six measurements for the C1 formulation and nine measurements for the C2 formulation.

Formulation	APS Measurement		Calculated *MMAD* (µm)
	*CMAD* (µm)	*σ*_g_	
C1	1.9 ± 0.1	1.7 ± 0.1	4.4 ± 0.4
C2	1.1 ± 0.1	1.5 ± 0.1	1.7 ± 0.2

A gently curved hose was used to connect the RBG-NOID outlet to the APS system in order to minimize deposition of large particles during transfer thereby shifting the measured size distribution to the lower size. For experiments on the C1 formulation, some deposition was seen within the tubing, indicating that despite precautions there was some particle deposition of the aerosol prior to reaching the APS system. Regardless, the *MMAD* of 4.4 ± 0.4 µm as measured at the outlet of the system demonstrated that the C1 formulation was close to target.

The APS device measures the particle size distribution of an aerosol by separating the particle measurements into bins before fitting the data to a lognormal distribution in order to calculate *CMAD* and σ_g_. The size range of the APS was 0.5–20 μm, with the lowest bin measuring all particles with aerodynamic particles <0.523 µm. Therefore, the *CMAD* reported by the APS system for the nose and lung C2 formulation may be higher than the actual *CMAD* of the powder. Regardless, the *MMAD* of 1.7 ± 0.2 µm as measured at the outlet of the system was within the 1–2 µm target for the C2 formulation.

### Powder Properties Post-processing

**FIGURE 3:**
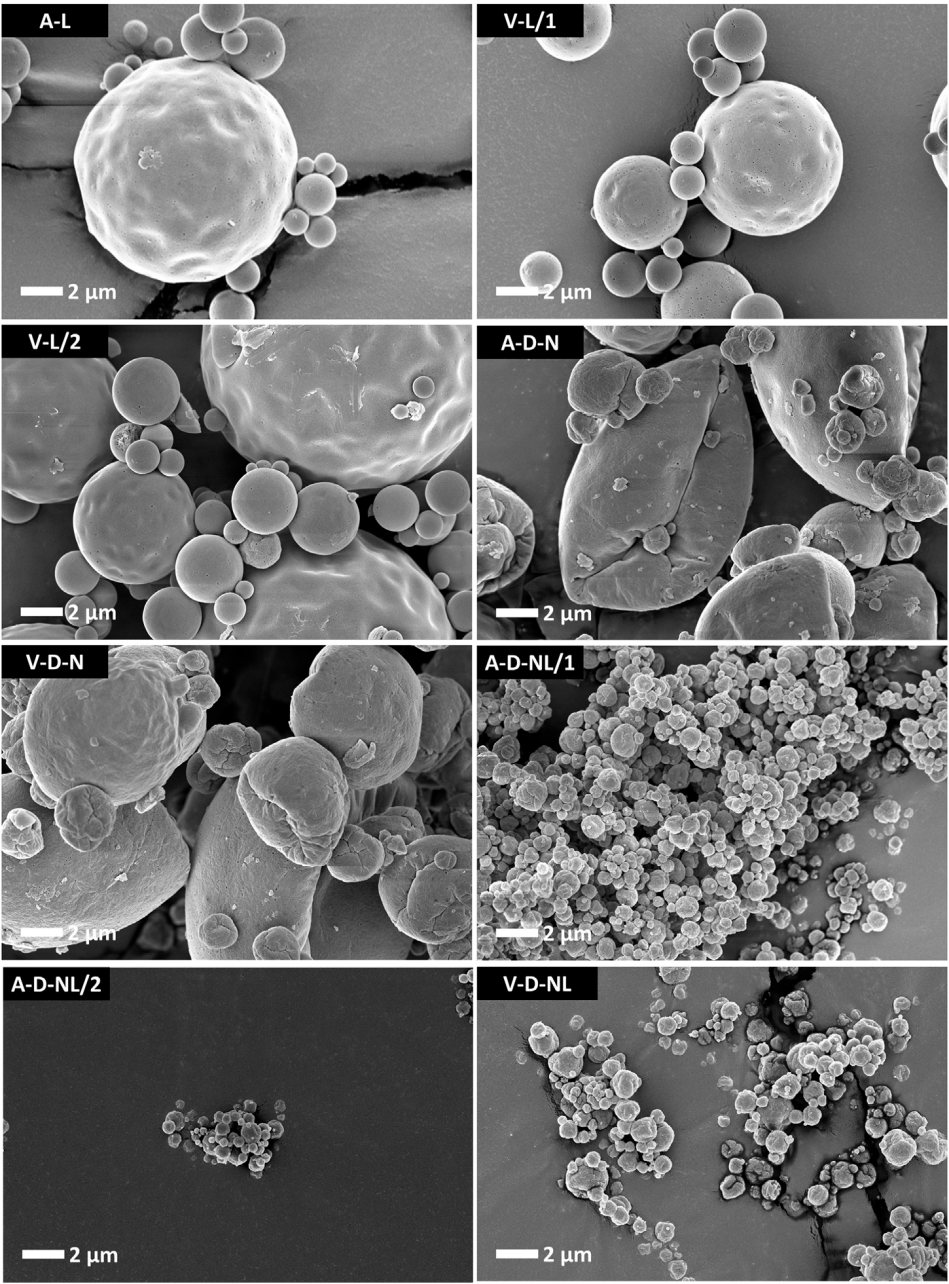
SEM images of the spray-dried powders prepared for the mouse model experiments. The lots containing trileucine that were designed for delivery of dry powder (A-D-N, V-D-N, A-D-NL/1, A-D-NL/2, and V-D-NL) demonstrate a more rugose particle morphology as compared to the lots intended for delivery after reconstitution (A-L, V-L/1, and V-L/2). The lots designed for dry powder deposition in the nose (A-D-N and V-D-N) are significantly larger than the lots designed for dry powder deposition in the nose and lungs (A-D-NL/1, A-D-NL/2, and V-D-NL). Smaller particles were designed to promote greater airway penetration. Scale bars are based on the respective images. Nomenclature: V–Vaccine; A–Adjuvant; L–Liquid; D–Dry powder; N–Nose; NL–Nose and Lung.

Particle morphology of the different lots manufactured for the mouse study are shown in Figure 3. Morphology for the different formulations was consistent with previous work spray drying the ID93+GLA-SE vaccine (Gomez et al., 2021a; Gomez et al., 2021b; Gomez et al., 2021c). Lots manufactured for IM or IN reconstituted liquid administration did not include trileucine as a dispersibility enhancer (A-L, V-L/1, and V-L/2) and consisted of round particles with overall smooth or lightly dimpled surfaces. Lots manufactured for dry powder aerosol delivery (A-D-N, V-D-N, A-D-NL/1, A-D-NL/2, and V-D-NL) have a more rugose outer particle surface. Of the lots designed for dry powder aerosol delivery, there is also a difference in size based on intended site of deposition. Lots designed for deposition in the nose and lungs (A-D-NL/1, A-D-NL/2, and V-D-NL) have a significantly smaller particle size than the lots designed for deposition in the nose (A-D-N and V-D-N). The former were designed to have a much lower overall solids content in the feedstock, leading to a smaller particle size.

**FIGURE 4:**
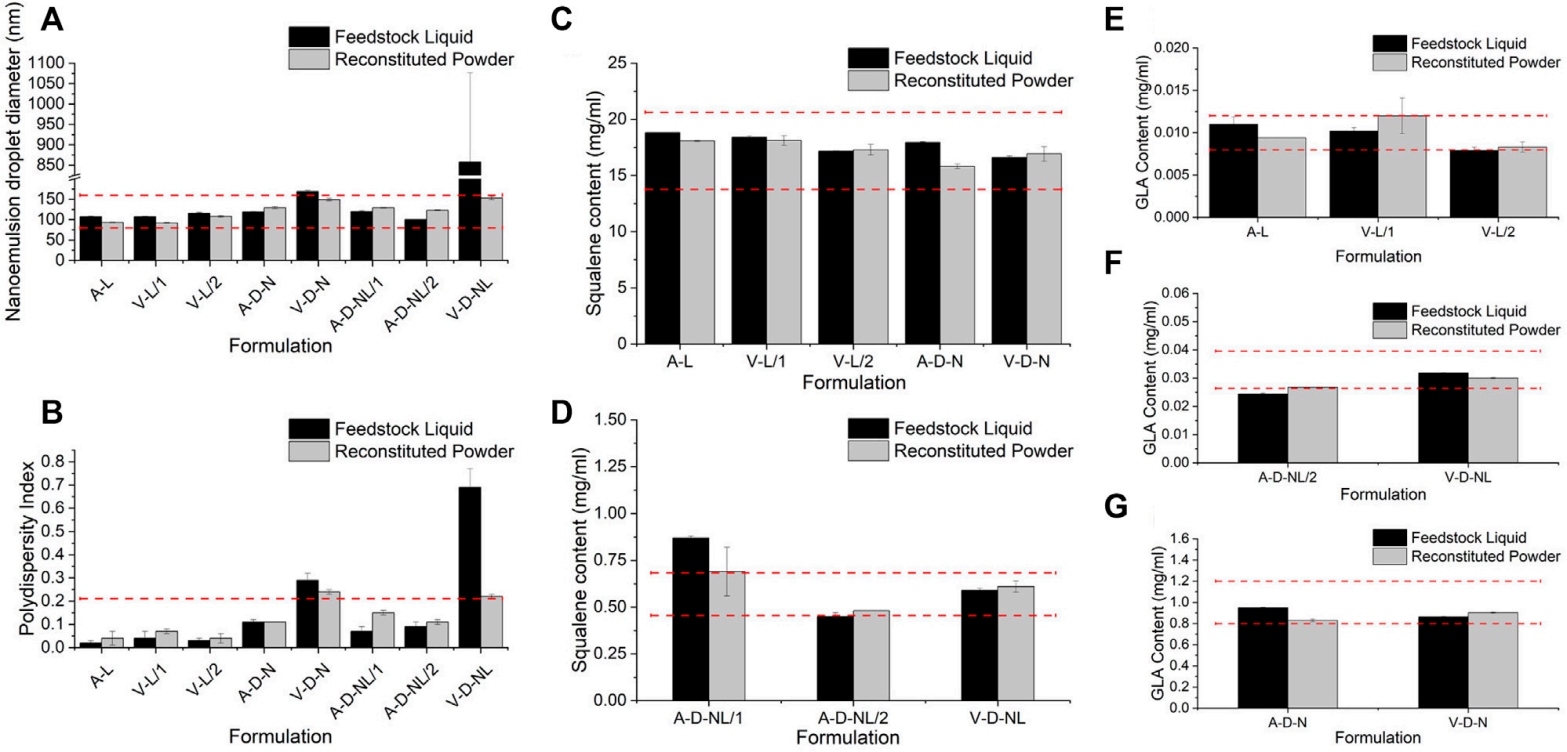
Comparison of the physicochemical properties before and after spray drying of the lots manufactured for the mouse model in terms of **(A)** nanoemulsion droplet diameter, **(B)** polydispersity index, **(C,D)** squalene content, and **(E–G)** GLA content. Measurements of the feedstock liquid are given in black, whereas measurements of the reconstituted powder are given in grey, for each lot. Results are reported as the mean ± standard deviation. The target range for each lot is demonstrated by the red dashed lines. Target range was a nanoemulsion droplet diameter measured as 120 ± 40 nm, polydispersity index <0.2, and squalene and GLA content ±20% of the target feedstock concentration. Nomenclature: V–Vaccine; A–Adjuvant; L–Liquid; D–Dry powder; N–Nose; NL–Nose and Lung.

Physicochemical properties of the spray-dried powder were assessed in terms of nanoemulsion droplet diameter, polydispersity index, squalene content, GLA content, and ID93 content. Due to the nature of these assays, it was necessary to reconstitute the spray-dried powders prior to analysis even for the formulations intended for dry powder delivery. Comparison of the adjuvant properties of the spray-dried powder to the feedstock liquid is shown in Figure 4. Target range was defined as nanoemulsion droplet diameter of 120 ± 40 nm, size polydispersity index <0.2, and squalene and GLA content ±20% of the target solution concentration (Table 3). Target range was based on acceptance criteria used in previous studies (Gomez et al., 2021a; Gomez et al., 2021c). The measured nanoemulsion droplet diameter, squalene content, and GLA content were within the target range for all spray-dried lots. The size polydispersity index of the V-D-N and V-D-NL lots was above the target range; however, this result may not be meaningful in practice since these two batches were intended for delivery as dry powders rather than reconstituted emulsion droplets.

The ID93 concentrations of the liquid feedstock and the reconstituted spray-dried powder of the vaccine-containing lots are given in Table 8. The spray-dried lots V-L/1 and V-L/2, which were intended for delivery upon reconstitution, were above and below the target ID93 concentration, respectively. These lots were mixed in a 1:1 ratio by mass to generate a single V-L lot for delivery with a calculated concentration of 0.0044 mg/ml, which was within the target range. All other measured vaccine properties were within the target concentration for both lots. The ID93 concentration of the spray-dried V-D-N lot was within target range. The ID93 concentration of the V-D-NL lot was just above target concentration; however, due to timeline and material constraints, this lot was deemed acceptable for the study.

**TABLE 8:** Comparison of the ID93 concentration of the liquid feedstock and the spray-dried powder as compared to the target concentration. Results are reported as the mean ± standard deviation. Nomenclature: V–Vaccine; L–Liquid; D–Dry powder; N–Nose; NL–Nose and Lung, ULOQ–Upper Limit of Quantitation (see Methods section).

Formulation	Target Range ID93 Concentration (mg/ml)	Liquid Feedstock ID93 Concentration (mg/ml)	Reconstituted Powder ID93 Concentration (mg/ml)
V-L/1	0.0032–0.0048	0.0059 ± 0.0012	0.0064 ± 0.0004
V-L/2	0.0032–0.0048	0.005	0.0023 ± 0.0001
V-D-N	0.32–0.48	0.57 ± 0.017	0.41 ± 0.008
V-D-NL	0.010–0.016	ULOQ	0.017 ± 0.0007

### Intramuscular Immunization With Reconstituted Spray-Dried ID93+GLA-SE Provides Similar Immunogenicity Profile as Reconstituted Lyophilized ID93+GLA-SE

To evaluate whether reconstituted spray-dried powder ID93+GLA-SE (Gomez et al., 2021a) maintained immunogenicity performance compared to the reconstituted lyophilized ID93+GLA-SE developed previously (Kramer et al., 2018), C57Bl/6 WT mice (n = 5/group) were IM immunized once with either formulation or with an excipient placebo containing trehalose in Tris buffer. One week following immunization, enumeration of tetramer-stained CD4^+^ T cells, cytokine production from intracellularly-stained CD4^+^ T cells, and serum antibody titers were measured. The reconstituted spray-dried formulation elicited a highly similar immunogenicity profile as the reconstituted lyophilized formulation (Supplementary Figure S1). Thus, in subsequent experiments, the reconstituted spray-dried ID93+GLA-SE administered IM was employed as a positive control to assess the immunogenicity and efficacy of the spray-dried ID93+GLA-SE delivered IN as a reconstituted powder, dry powder nasal delivery via large particle aerosol, or dry powder pulmonary delivery via small particle aerosol.

### Effect of Vaccine Delivery on Cellular Immune Responses in the Lung and Spleen

**FIGURE 5:**
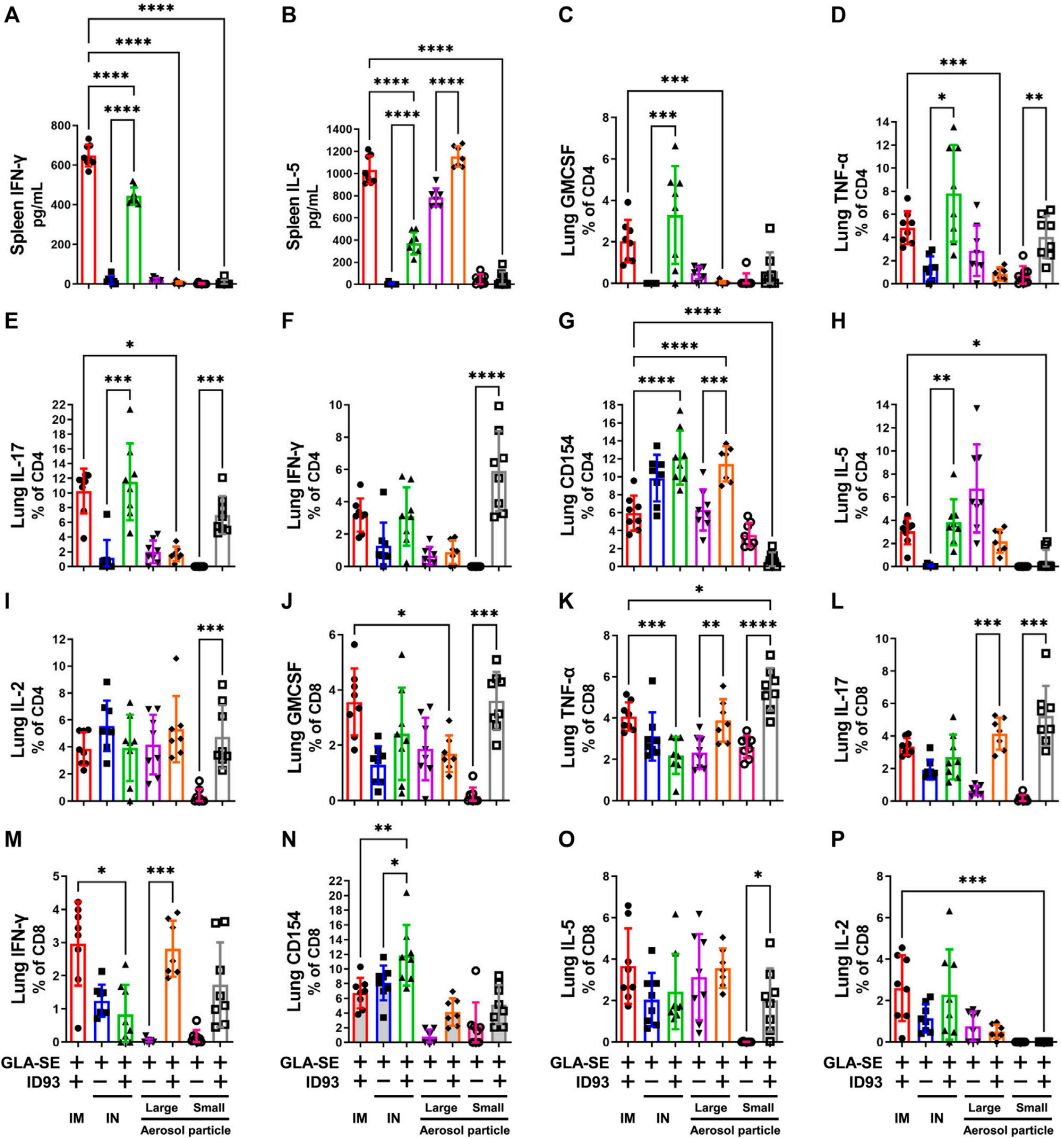
IM, IN, and aerosol vaccinations with ID93+GLA-SE induce cellular immune responses in vaccinated mice. B6 (n = 7–8) mice were either vaccinated with ID93+GLA-SE or only GLA-SE through intramuscular (IM), intranasal (IN), nasal aerosol delivery of large particle dry powder, or pulmonary aerosol delivery of small particle dry powder, at day 0 and day 21. Mice spleens were harvested at 4 weeks post final immunization and stimulated with ID93 antigen (2 μg/ml for 48 h). Levels of splenic **(A)** IFN-γ and **(B)** IL-5 were detected in the supernatant by ELISA. Splenocyte ELISA data shown are from one experiment, and similar patterns of response were evident in the repeat experiment. Frequency of **(C)** CD4^+^GMCSF^+^, **(D)** CD4^+^TNF-α^+^, **(E)** CD4^+^IL-17^+^, **(F)** CD4^+^IFN-γ^+^, **(G)** CD4^+^CD154^+^, **(H)** CD4^+^IL-5^+^, **(I)** CD4^+^IL-2^+^, **(J)** CD8^+^GMCSF^+^, **(K)** CD8^+^TNF-α ^+^, **(L)** CD8^+^IL-17^+^, **(M)** CD8^+^IFN-γ^+^, **(N)** CD8^+^CD154^+^, **(O)** CD8^+^IL-5^+^, and **(P)** CD8^+^IL-2^+^ T cells in the lung were detected using flow cytometry. Mice lungs were harvested at 4 weeks post final immunization for detection of immune cell components. Flow cytometry data shown are from one experiment and were not repeated in the second mouse experiment. For both flow cytometry and ELISA readouts, responses in unstimulated controls were subtracted from the stimulated samples, and any resulting negative values were assigned as zero. *p < 0.05, **p < 0.01, ***p < 0.001, and *p* < 0.0001 by one-way ANOVA or Welch’s ANOVA with Sidak’s or Dunnet’s T3 correction, respectively, for multiple comparisons between selected groups. The Kruskal-Wallis non-parametric test with Dunn’s correction for multiple comparisons was used when multiple comparisons were not possible with Welch’s ANOVA due to one experimental group having constant values.

Cytokine secretion by the host immune cells is necessary to control *Mtb* infection as well as *Mtb* induced pathogenesis. Previous studies have shown that IM immunization with ID93+GLA-SE elicits a robust Th1 response in the spleen and lung and protects upon Mtb challenge in mice and guinea pigs (Orr et al., 2015). In contrast, IN immunization switched this response to a Th17 response with significant production of IL-17, but not IFN-γ, by CD4^+^ T cells in the spleen and lung (Orr et al., 2015). Here, we determined the ability of the different vaccine delivery approaches to induce cytokine responses in the spleen and lung. The immunizations were administered, as outlined in Table 1, to C57BL/6 WT mice (14–16 mice per group) with reconstituted spray-dried ID93+GLA-SE or GLA-SE alone (IM or IN delivery), or ID93+GLA-SE or GLA-SE alone in spray-dried powder form (large or small particles delivered via aerosol route). No significant induction of IL-17 or IL-5 cytokine in the *ex vivo* stimulated splenocytes was observed for any experimental group at 1 week post second immunization, and induction of IFN-γ was only observed in splenocytes from mice vaccinated IM with reconstituted ID93+GLA-SE (data not shown). At 4 weeks post-immunization, IL-17 levels remained below detection, whereas IFN-γ (Figure 5A) and IL-5 (Figure 5B) were measured in mice vaccinated IM or IN with reconstituted ID93+GLA-SE. Significantly increased levels of IL-5 in the supernatants were also observed from the ID93+GLA-SE large particle nasal aerosol vaccinated group compared to adjuvant alone.

ID93 antigen-specific cellular responses in the lung were assessed 4 weeks post immunization. IM immunization with ID93+GLA-SE elicited appreciable levels of a diversity of markers including GMCSF, TNF-α, IL-17, IFN-γ, and IL-5 (Figures 5C–I). Significantly higher induction of GMCSF, TNF-α, IL-17, and IL-5 expressing CD4^+^ T cell frequencies was observed in mice vaccinated IN with reconstituted ID93+GLA-SE compared to control mice receiving adjuvant alone. Interestingly, the only significantly upregulated CD4^+^ T cell marker in mice immunized with large particle ID93+GLA-SE nasal aerosol was CD154. In contrast, mice immunized with small particle ID93+GLA-SE pulmonary aerosol generated significant levels of CD4^+^ T cells expressing TNF-α, IL-17, IFN-γ, and IL-2. We also determined the immune cell activation in the CD8^+^ T cell compartment. Like the CD4^+^ T cell compartment, the data showed increased frequency of GMCSF, TNF-α, and IL-17 expressing CD8^+^ T cells in the lungs of ID93+GLA-SE small particle aerosol vaccinated groups compared to their respective control mice 4 weeks post immunization (Figure 5J–P). Interestingly, higher frequency of IL-5 expressing CD8^+^ T cells in the lungs of ID93+GLA-SE small particle aerosol vaccinated group was also shown as compared to the control mice. In addition, significantly increased frequency of TNF-α, IL-17, and IFN-γ expressing CD8^+^ T cells was observed in the lungs of ID93+GLA-SE large particle nasal aerosol vaccinated groups compared to their respective control mice 4 weeks post immunization. However, unlike CD4^+^ T cells, there was no significant induction of these populations in the lungs of mice vaccinated IN with reconstituted ID93+GLA-SE when compared with their respective control mice.

Some CD8^+^ and CD4^+^ T cell activity was detected even in the adjuvant-alone groups in some readouts; therefore, the response magnitude of vaccinated groups was interpreted with reference to the background signal in groups receiving adjuvant alone. The production of measurable CD8^+^ T cell responses as well as IL-5 by splenocytes and CD4^+^ T cells in the lung following IM or IN immunization of liquid ID93+GLA-SE are not consistent with our previous experience (Orr et al., 2015; Kramer et al., 2018); the cause for these discrepancies is unclear. Nevertheless, the data suggested that reconstituted IM, reconstituted IN, and both aerosol formulations induced differential antigen-specific cellular immune response profiles in the lung, whereas the small particle pulmonary aerosol formulation did not elicit measurable cytokine responses in the spleen.

### Effect of Vaccine Delivery Method on Antibody Responses

**FIGURE 6:**
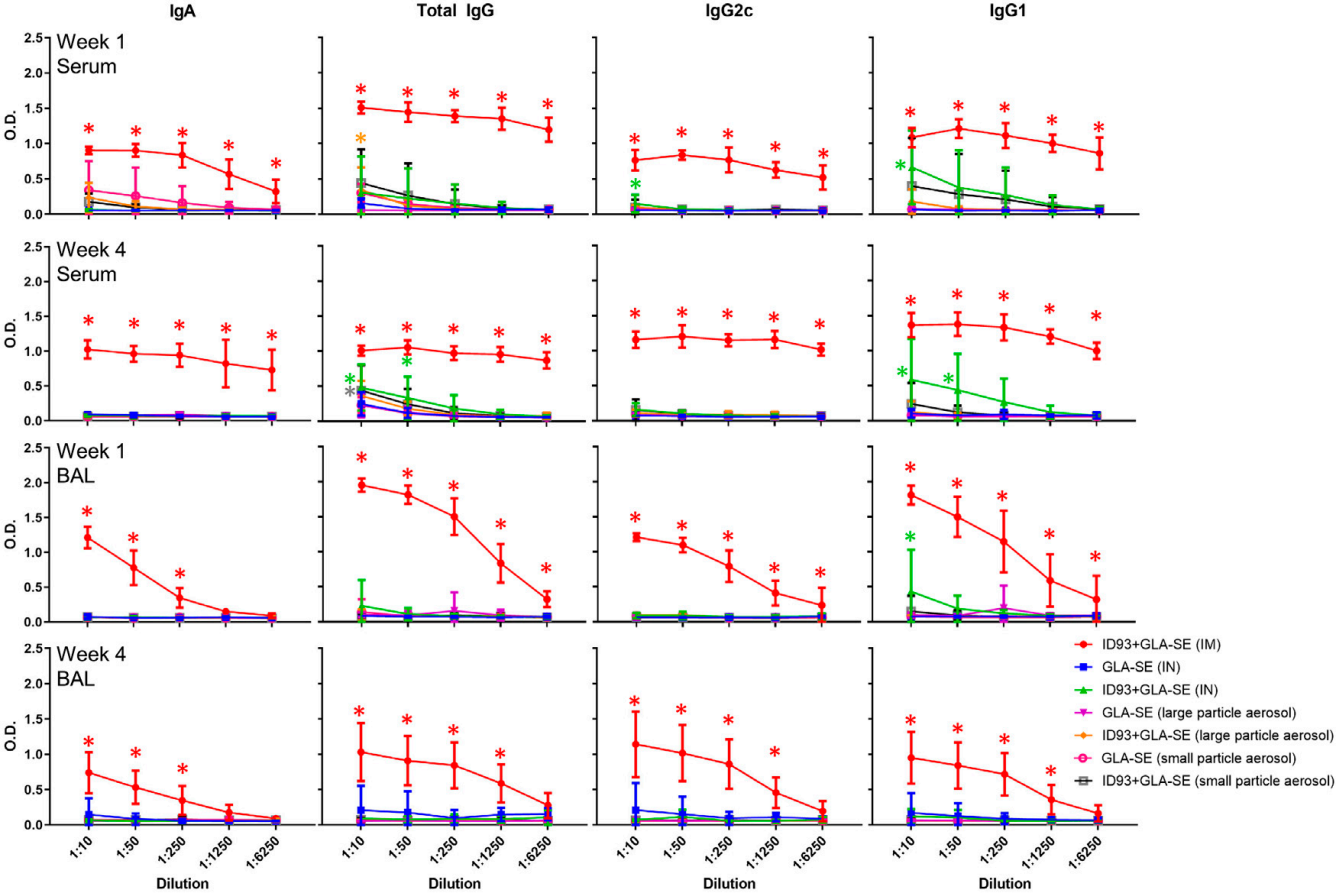
IM vaccination with ID93+GLA-SE induces greater serum and mucosal antibody responses than alternative routes/presentations. B6 (n = 7–8) mice were vaccinated with ID93+GLA-SE or only GLA-SE through intramuscular (IM), intranasal (IN), nasal aerosol delivery of large particle dry powder, or pulmonary delivery of small particle dry powder, at day 0 and day 21. Antigen-specific IgA, total IgG, IgG2c, and IgG1 antibody responses in serum and bronchoalveolar lavage (BAL) were measured 1 and 4 weeks following the second immunization by ELISA. Data presented as mean ± SD. For simplicity, all statistical differences are represented as *p < 0.05 even when lower *p*-values were achieved. Each group immunized by IN or aerosol routes is compared to their adjuvant alone controls. The IM-immunized group comparison (red asterisk) represents statistical significance compared to all 3 alternative routes/presentations of ID93+GLA-SE. Statistical evaluation was conducted by two-way ANOVA with Tukey’s correction for multiple comparisons between selected groups.

We measured the levels of antigen-specific antibodies in the serum following immunization and observed that mice that were vaccinated IM with reconstituted ID93+GLA-SE had the highest magnitude of antibody titers for all isotypes measured at both 1 and 4 weeks post immunization (Figure 6). Serum total IgG, IgG2c, and IgG1 levels were slightly elevated in mice vaccinated IN with reconstituted ID93+GLA-SE compared to the adjuvant alone control, and serum total IgG was likewise somewhat increased in aerosol vaccine groups compared to adjuvant alone controls. Nevertheless, no alternative immunization route/presentation approached the level of serum antibodies elicited by IM immunization. We also determined antigen-specific antibody titers in the BAL samples from immunized animals. Interestingly, we observed substantially enhanced antibody titers in mice immunized IM with reconstituted ID93+GLA-SE at both 1 and 4 weeks post immunization, but there was no significant elevation of BAL antibodies in the experimental groups with the alternative routes/presentations with the exception of slightly increased IgG1 titers in the IN-immunized group 1 week following the second immunization. Finally, to determine the induction of long-lived B cell specific antibody responses following immunization, we harvested and analyzed the bone-marrow tissues (BM) of immunized animals at 1 and 4 weeks post immunization using antigen-specific ELISpot assay. Long-lived IgA and IgG-secreting responses were consistently detected in the BM cells from mice immunized IM with reconstituted ID93+GLA-SE at both time points but not in the BM cells from mice in the other experimental groups (Supplementary Figure S3). Overall, none of the alternative routes/presentations approached the level of serum antibodies, mucosal antibodies, or long-lived antibody-secreting cells in the bone marrow induced by IM immunization. Therefore, our data suggested an antibody-independent mechanism of protection for mice immunized by alternative routes/presentations of ID93+GLA-SE.

### Aerosol Delivery of Small Particle Size Spray-Dried ID93+GLA-SE Confers Protection in a Mouse Model of *Mtb*

**FIGURE 7:**
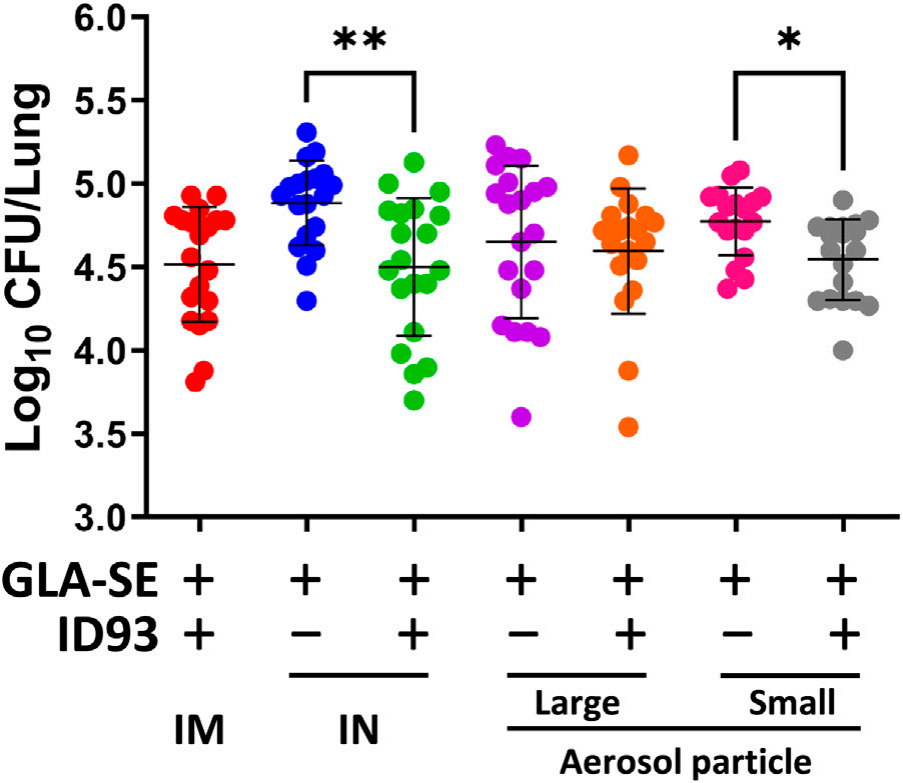
IN delivery of reconstituted liquid ID93+GLA-SE or pulmonary aerosol delivery of small particle size spray-dried powder ID93+GLA-SE confers protection in a mouse model of *Mtb*. B6 mice (n = 18–20) were vaccinated with ID93+GLA-SE or only GLA-SE through reconstituted liquid intramuscular (IM), reconstituted liquid intranasal (IN), nasal aerosol delivery of large particle dry powder, or pulmonary delivery of small particle dry powder, at day 0 and day 21. All groups of B6 mice were rested for 4 weeks after which mice were challenged with *Mtb* H37Rv (100 CFU). *Mtb* CFU was determined at 4 weeks post-infection. Data presented are combined results from two identical experiments, showing mean ± SD. *p < 0.05 and **p < 0.01 by one-way Welch’s ANOVA with Dunnett’s T3 correction for multiple comparisons between selected groups.

IM immunization with liquid or reconstituted lyophilized ID93+GLA-SE elicits a robust Th1 response and limits Mtb in animal efficacy models (Kramer et al., 2018). Furthermore, we previously demonstrated that IN immunization with liquid ID93+GLA-SE is as efficacious as the IM route of the vaccine (Orr et al., 2015). The immunizations were administered, as outlined in Table 1, to C57BL/6 WT mice (8–10 mice per group) with reconstituted spray-dried ID93+GLA-SE or GLA-SE alone (IM or IN delivery), or ID93+GLA-SE or GLA-SE alone in spray-dried powder form (large or small particles delivered via aerosol route), followed by challenge with *Mtb* H37Rv. The lung bacterial burden following pulmonary aerosol delivery of smaller particle size spray-dried ID93+GLA-SE was observed to be significantly lower than with the adjuvant alone (Figure 7). The level of protection in the lungs derived from the smaller particle size spray-dried powder ID93+GLA-SE was comparable to the reconstituted vaccine delivered as a liquid via the IM or IN routes of delivery. Immunization via nasal aerosol delivery of the larger particle size spray-dried powder ID93+GLA-SE did not cause a reduction in lung bacterial burden compared to adjuvant alone. However, the level of mean lung CFUs among the four experimental groups administered ID93+GLA-SE was similar and, for the statistically significant differences shown in Figure 7, represented a 0.2–0.4 log reduction in CFUs compared to the experimental groups administered GLA-SE alone. In our previously published experiments using similar mouse challenge models with lyophilized ID93+GLA-SE or other recombinant protein or DNA vaccines, immunized animals tended to reduce lung CFU burden by 0.4–1 log compared to unvaccinated controls, where mean log CFU levels in the lung for control mice were ∼4.7–5.3 for this challenge system (Orr et al., 2013; Gopal et al., 2014; Orr et al., 2014; Griffiths et al., 2016b; Ahmed et al., 2017a; Ahmed et al., 2017b). There were no significant differences observed in spleen bacterial burden among the groups of mice infected with *Mtb,* although the lowest mean bacterial burden was associated with spray-dried pulmonary aerosol delivery (Supplementary Figure S4). In summary, these results demonstrated that the spray-dried pulmonary aerosol vaccine delivery and the liquid IN vaccine delivery resulted in significant protective efficacy against *Mtb* challenge compared to adjuvant alone delivered by the same route.

## Discussion

Pulmonary TB continues to persist as a global pandemic due to the lack of an effective human vaccine for over a hundred years since the implementation of the only licensed BCG vaccine (Colditz et al., 1994). Complications from the current COVID-19 pandemic in combination with the already existing emergence of drug-resistant strains of Mtb have further heightened the spectre of TB. The respiratory tract is the natural route of Mtb infection. Therefore, when compared with traditional parenteral routes of vaccination, there is general agreement that mucosal vaccination induces superior protection against Mtb challenge (Goonetilleke et al., 2003; Chen et al., 2004; Wang et al., 2004; Santosuosso et al., 2005; Perdomo et al., 2016; Mata et al., 2021; Ning et al., 2021). The successful formulation and manufacture of the prophylactic TB vaccine (ID93) co-lyophilized with the GLA-SE oil-in-water emulsion adjuvant produced a thermostable, single-vial candidate product that is being evaluated in an ongoing clinical trial as a reconstituted IM vaccine (Kramer et al., 2018). We further developed the same vaccine as a thermostable spray-dried powder to aid in bulk storage and mucosal delivery for improved utility in clinical field applications (Gomez et al., 2021a; Gomez et al., 2021b; Gomez et al., 2021c). In this study, we evaluated the immunogenicity and efficacy of the spray-dried ID93+GLA-SE powder delivered as an inhaled aerosol either to the nose and lungs (pulmonary delivery) using smaller particle size spray-dried formulations, or to the nose (nasal delivery) using larger particle size spray-dried formulations, compared to the reconstituted liquid vaccine administered intranasally or by the conventional intramuscular route.

Development and characterization of an aerosol system is critical for successful vaccine-inhalation animal studies. Optimization experiments with the vehicle-only powder formulations demonstrated that delivery efficiency was improved with reduced powder feed rate. The small particle C2 formulation, targeting deposition in the nose and lungs of mice, achieved a much higher system efficiency than the larger particle C1 formulation, which targeted deposition in the nose only. Higher system efficiency of the former is likely due to the smaller particle size as larger particles are expected to deposit due to inertial impaction or sedimentation as the aerosol traverses the aerosol system. Optimization results also demonstrated that the constrictions on tolerable duration of exposure and aerosol concentration at the noseports limited the possible amount of powder that could be delivered to the mice. Therefore, the active components of experimental vaccines needed to be concentrated above intended clinical dosage to achieve the target dose in preclinical aerosol studies.

The system efficiencies of the small and large particle vehicles are a clear improvement over a recent study that delivered nebulized bacteriophage-containing droplets to mice using an aerosol system consisting of a vibrating mesh nebulizer and the NOID characterized in this study (Carrigy et al., 2019). Those experiments were run with an aerosol flow rate of 500 ml/min and the overall system efficiency was only 1.50% of the nominal dose measured on the exit filter and 0.033% of the nominal dose measured at the noseports under optimized processing parameters (Carrigy et al., 2019). Conversely, the current study demonstrated that 8.9 and 40.0% of the nominal dose were measured on the exit filter for the large particle vehicle and small particle vehicle, respectively. Improved system efficiency is likely due to the engineered properties of the particles that resulted in high aerosol performance, as well as possible re-entrainment within the system.

A lower delivered dose relative to the nominal dose was measured in this study, with approximately 0.024 and 0.11% estimated to be inhaled at the noseports for the small particle vehicle and large particle vehicle under optimized conditions, respectively. However, this low relative nominal dose can be attributed to the necessary higher aerosol flow rate of 8.33 L/min required to operate the aerosol delivery device used in this study. Due to the difference between aerosol flow rate and the total inhalation rate of the mice, approximately 97% of aerosol available will bypass the mice. Consequently, even in an ideal system with no losses, the maximum dose delivered to each mouse in a 12-mouse experimental set up would be only 0.25% of the nominal dose. Therefore, delivered dose was approximately 10 and 44% for the C1 and C2 formulations under optimized conditions, respectively, relative to the maximum possible delivered dose. The difference in the estimated delivered dose versus maximum possible dose was accounted for by engineering higher concentrations of active ingredients ID93 and GLA to ensure delivered doses of these components were approximately equivalent regardless of route. Nevertheless, it is possible that the actual delivered dose of antigen and adjuvant varied from these estimates, which could impact the immunogenicity and efficacy results.

The size distribution of the C1 and C2 powders measured at the outlet of the aerosol system was close to the target of ∼5 μm and 1–2 μm, respectively. These dispersion results suggest that the chosen operating parameters of a high brush speed, low powder feed flow rate, and low aerosol flow rate were suitable for dispersing the powder. However, the powders tested in this study were designed to be easily dispersible due to the accumulation of trileucine on the surface. System efficiency and dispersion capability is strongly dependent on the particle size, surface composition, and surface morphology.

Previous work developing spray-dried versions of the ID93+GLA-SE vaccine (Gomez et al., 2021a; Gomez et al., 2021b; Gomez et al., 2021c) led to successful manufacturing of the formulations for the mouse model. The formulations designed for delivery upon reconstitution were prepared without trileucine whereas the formulations designed for aerosolized delivery included trileucine as a dispersibility enhancer. Further discussion on the inclusion of a dispersibility enhancer to improve aerosol performance of a powder can be found elsewhere (Gomez et al., 2021b). The powders without trileucine formed smooth particles primarily composed of trehalose. These powders are expected to exhibit poor aerosol performance due to their smooth surface morphology. By contrast, the trileucine-containing particles demonstrated rugose surface morphology. This change in surface morphology due to the inclusion of trileucine has been previously shown to improve aerosol performance (Gomez et al., 2021b). The nanoemulsion diameter, size polydispersity index, squalene content, and GLA content were within target for all spray-dried formulations with the exception of the V-D-N and V-D-NL lots that were above the size polydispersity index target. These lots both include trileucine and the ID93 antigen. Previous work has suggested that inclusion of trileucine increases nanoemulsion droplet diameter and polydispersity index (Gomez et al., 2021c). However, the acceptance criteria for polydispersity index were originally based on a dosage form designed for reconstitution and injectable delivery (Kramer et al., 2018), and it is likely that this parameter is not meaningful for an inhaled administration route that does not require reconstitution. A more important parameter to address is the antigen content since ID93 processing loss was variable, indicating that further work is needed to optimize spray drying of the ID93 antigen.

When compared to systemic routes of immunization, mucosal vaccines induce better immunity and confer superior protection against mucosal infectious diseases, including TB (Goonetilleke et al., 2003; Chen et al., 2004; Wang et al., 2004; Santosuosso et al., 2005; Neutra and Kozlowski, 2006; Perdomo et al., 2016; Ahmed et al., 2017a; Ahmed et al., 2017b). Mucosal delivery of vaccines promotes both systemic and mucosally localized adaptive immune responses, whereas parenteral immunization primarily programs systemic immunity. BCG or MVA85A (or as a booster to BCG) delivered by intradermal injection both elicit Th1 responses (Tameris et al., 2014). The gold-standard vaccine BCG protects against disseminated childhood TB, but protection against lung TB in adolescents and adults is variable and mostly poor. MVA85A administered as a booster to BCG was safe but not effective in reducing the risk of developing TB (Tameris et al., 2013; Kashangura et al., 2019). Interestingly, intranasal BCG vaccination has been reported to provide short-term enhancement of protection in the lung relative to subcutaneous immunization, with potent and extremely persistent splenic protective responses lasting for 10 months following respiratory immunization in mice (Derrick et al., 2014). Higher frequencies of CD4^+^ T cells expressing gamma interferon (IFN-γ) and IFN-γ/TNF-α, as well as CD8^+^ T cells expressing IFN-γ, were detected in the spleens of intranasal BCG vaccinated mice. Recent studies in macaques have shown that mucosal or intravenous BCG better protects rhesus macaques from Mtb infection and TB disease than standard intradermal vaccination, correlating with local adaptive immune signatures (Vierboom et al., 2021). Moreover, Th17 cells that produce IL-17 play a critical role as primary effector cells mediating vaccine-induced protection against *Mtb* (Khader et al., 2007; Gopal et al., 2013; Griffiths and Khader, 2014; Aguilo et al., 2015; Monin et al., 2015; Counoupas et al., 2020). In the current study, we found that the pulmonary aerosol delivery of the spray-dried vaccine provided protection against *Mtb* challenge compared to adjuvant alone. The pulmonary-delivered vaccine also elicited increased levels of both IFN-γ and IL-17 responses by CD4^+^ T cells in the lung compartment; however, it did not elicit appreciable serum or mucosal antibody responses. Indeed, little or no antibody responses were elicited in all groups immunized by the mucosal routes, possibly indicating antigen degradation or conformational alteration following mucosal administration. While the role of antibodies in protection against *Mtb* is unclear (Jacobs et al., 2016), the importance of cell-mediated immunity is well-established. Although immunization via nasal aerosol delivery of the larger particle size spray-dried powder ID93+GLA-SE did not cause a reduction in lung bacterial burden compared to adjuvant alone, it is unclear whether the lung CFU levels of the adjuvant alone group in this case were associated with a non-specific protective effect or whether this could be attributable to experimental variation.

In previous studies, we have demonstrated that localization of vaccine-induced CD4^+^ T cells within parenchyma and activation of myeloid cells promoted the formation of protective lymphoid-containing granuloma structures within the lung and the control of *Mtb* replication (Das et al., 2021). *Mtb* is considered as a successful pathogen for its ability to escape host immune responses efficiently. Studies have demonstrated that, following *Mtb* infection, delay in the activation of antigen-specific CD4^+^ T cell responses occurs likely due to *Mtb*’s ability to directly inhibit MHC-II transactivator expression, MHC–II expression, and antigen presentation (Harding and Boom, 2010). BCG vaccination can generate systemic vaccine-induced T cell responses. Our recent work demonstrates that rapid amplification of early CD4^+^ T cell response and localization within airway and parenchyma compartments in the lung is necessary for superior vaccine induced immunity (Das et al., 2021). Similar response is seen in non-human primates vaccinated intravenously with BCG, where the heightened and lung-localized T resident memory (Trm) cells are considered to be a mechanism through which complete control of *Mtb* infection is mediated (Darrah et al., 2020). Based on our current study, we expect that delivery of spray-dried IN and dry powder aerosol vaccinations can induce activation of antigen-specific T cells in the lymph nodes and possibly recruitment to the lung. Thus, our results, along with recent reports including intravenous (Darrah et al., 2020) and mucosal BCG use (Verreck et al., 2017), suggest that generating a lung-resident activated T cell pool is a good strategy for improving vaccine-induced immunity against TB. However, we also consider the possibility of local proliferation of T cells following aerosol vaccination in this study.

During *Mtb* infection, recognition of infected macrophages in the lung by CD4^+^ effector T cells is required for intracellular *Mtb* control (Srivastava and Ernst, 2013). Therefore, localization of Trm CD4^+^ T cells in the lung is an important event required for *Mtb* control. In human studies, CXCR5^+^ CCR5^+^ T cells in the lungs and pleural fluid produced IFN-γ (Lindestam Arlehamn et al., 2013; Perreau et al., 2013; Saha et al., 2013), a signature Th1 response. Moreover, in the preclinical macaque model of latent and active TB, CXCR3^+^CCR6^+^ co-expressing T cells produced both IL-17 and IFN-γ cytokines in the BAL and were associated with the protective responses in latent TB (Shanmugasundaram et al., 2020). Finally, in vaccine models of subunit vaccination, CD4^+^ T cells that were able to readily traffic to the lung parenchyma, provided *Mtb* control *in vivo* (Woodworth et al., 2017). Both IFN-γ and IL-17 have different roles in controlling *Mtb* following vaccination. While recent data have shown that IFN-γ (Gopal et al., 2013) and IFN-γ produced by CD4^+^ T cells (Sallin et al., 2017) are considered redundant, IL-17 is necessary for vaccine-induced control in many models of vaccination (Khader et al., 2007; Gopal et al., 2012; Kumar et al., 2016). More recent work from our lab has also demonstrated a critical combined role for IL-17/IL-22 and IFN-γ in conferring early vaccine-induced control of *Mtb* infection (Das et al., 2021). Therefore, it is likely that the mucosal delivery of vaccine to the lung can accelerate parenchymal homing of antigen-specific CD4^+^ T cell subsets to gain access to the *Mtb*-infected cells within the granuloma and reduce *Mtb* replication through activation of signaling involving both the Th1/Th17 cytokine axis along with other mediators.

There are several limitations to the current study. Although tetramer-based staining and draining lymph node T cell responses were evaluated in the initial immunogenicity experiment (Supplementary Figure S1), subsequent immunogenicity experiments did not include tetramer-based staining or assess lymph node activity due to practical limitations such as experimental throughput and cell viability based on the number of mice and other assays to be performed. For similar considerations, and based on previous experience that lung bacterial burden in animals administered saline alone was indistinguishable from lung bacterial burden in animals administered GLA-SE alone (Bertholet et al., 2010), saline or non-vaccinated control groups were not included in the protective efficacy experiments reported here. Nevertheless, it cannot be ruled out that GLA-SE administered by alternative routes results in non-specific effects in the efficacy results shown here.

## Conclusion

The administration routes of IM injection, IN delivery of a liquid, IN delivery of a dry powder, and pulmonary delivery of a dry powder, was compared within a mouse model for the spray-dried TB vaccine ID93+GLA-SE. A custom-built aerosol delivery system was used for the inhalation component of the study. This system consisted of two main components: a dust generator to aerosolize the spray-dried vaccine and a nose-only inhalation device to restrain the mice and deliver the aerosolized dry powder vaccine. The aerosol delivery system parameters were optimized using two control formulations: 1) a formulation that would target nose-only deposition and 2) a formulation that would target nose and lung deposition in mice. Experiments with both formulations showed that the designed system greatly outperformed the aerosol delivery efficiency of a similar set up and was capable of dispersing the spray-dried powder to a particle size suitable for inhalation by mice.

The pre-clinical experiment design consisted of seven experimental groups that would immunize mice with either the spray-dried vaccine or an adjuvant-alone spray-dried powder as a negative control. These experiments were also designed to compare different routes of delivery, namely, IM injection, IN delivery of a reconstituted liquid, nasal delivery of a dry powder, and pulmonary delivery of a dry powder. Different formulations and spray drying parameters were used for manufacturing the powder for the different experimental groups based on the route of delivery. Analysis of the powder found that particle morphology for each lot was as expected. The physicochemical integrity of all formulations was also assessed after spray drying in terms of nanoemulsion droplet size distribution, squalene content, GLA content, and ID93 content. Results showed that the different formulations were either within or close to target, demonstrating that spray drying is a viable method of vaccine desiccation. This conclusion was further confirmed through the similar immune responses generated by the reconstituted spray-dried vaccine to the reconstituted lyophilized presentation when administered IM in a mouse model.

Our results show that both IN liquid vaccine delivery as well as pulmonary dry powder vaccine delivery resulted in *Mtb* control in infected mice. Additionally, improved protection in these two vaccinated groups over their respective control groups coincided with the presence of cytokine-producing T cell responses, whereas antibody responses in all groups were substantially reduced compared to IM immunization. Our results demonstrate that spray-dried ID93+GLA-SE administered by IM or alternative routes elicits protective efficacy against *Mtb* infection. While pulmonary dry powder vaccine delivery did not improve upon the levels of protective efficacy of the liquid vaccine administered IM or IN, the dry powder formulation offers practical advantages including enhanced stability and simplicity of administration that does not require needles nor reconstitution. Even for reconstituted injectable vaccines, a spray-dried powder may offer cost and scalability advantages compared to a lyophilized cake (Gomez et al., 2021a). In addition, these results highlight the importance of a rational approach based on formulation and engineering principles for the development of spray-dried powder vaccines, taking into account the practical limitations and complexity of testing such formulations in small animal experimental models. Finally, these results motivate further development of spray-dried ID93+GLA-SE powder to assess immunogenicity and efficacy in large animal models with greater relevance to humans in terms of anatomy and Toll-like receptor expression.

## Data Availability

The raw data supporting the conclusions of this article will be made available by the authors, without undue reservation.
