# Supplementary material for: Development and Testing of a Spray-Dried Tuberculosis Vaccine Candidate in a Mouse Model
Source: Front Pharmacol. 2022 Jan 21;12:799034. doi: 10.3389/fphar.2021.799034 (PMC8814656; doi:10.3389/fphar.2021.799034)
Supplement: Supplementary file 1 [file DataSheet1.pdf]

Supplementary Table 1. Flow cytometry antibody panels.

**Immunogenicity Experiment in Supplementary Figure 1: Intracellular cytokine panel**				
Antibody	Fluorochrome	Dilution	Clone	Manufacturer
GM-CSF	FITC	1:100	MPI-22E9	BioLegend
CD154	PerCP-710	1:100	MR1	eBioscience
TNF	eF450	1:100	MP6-XT22	eBioscience
CD8<br>B220<br>CD11b	BV510	1:200<br>1:200<br>1:200	53-6.7<br>RA3-6B2<br>M1/70	BioLegend<br>BioLegend<br>BD Biosciences
CD4	BV650	1:200	RM4-5	BioLegend
IL-2	APC	1:100	JES6-5H4	eBioscience
IL-17A	Alexa700	1:100	TC11-18H10	BioLegend
CD44	APC-eF780	1:200	IM7	eBioscience
IL-5	PE	1:100	TRFKS	BioLegend
IFNγ	PE-Cy7	1:100	XMG1.2	eBioscience
**Immunogenicity Experiment in Supplementary Figure 1: CD4 T cell tetramer stain panel**				
Antibody	Fluorochrome	Dilution	Clone	Manufacturer
FoxP3	Alexa488	1:100	FJK-16s	eBioscience
CXCR5	PerCP-710	1:200	SPRCL5	eBioscience
T-bet	BV421	1:100	4B10	BioLegend
CD8<br>B220<br>CD11b	BV510	1:200<br>1:200<br>1:200	53-6.7<br>RA3-6B2<br>M1/70	BioLegend<br>BioLegend<br>BD Biosciences
PD-1	BV605	1:200	29F.1A12	BioLegend
CD4	BV650	1:200	RM4-5	BioLegend
CD44	Alexa700	1:200	IM7	eBioscience
**Immunogenicity Experiment in Figure 5: Intracellular cytokine panel**				
Antibody	Fluorochrome	Dilution	Clone	Manufacturer
GM-CSF	FITC	1:100	MPI-22E9	BioLegend
CD154	PerCP-Cy5.5	1:100	MR1	eBioscience
TNF	Pacific Blue	1:100	MP6-XT22	eBioscience
Live dead	BV605	1:1000	-	Thermo Fisher Scientific
CD8	BV510	1:200	53-6.7	BioLegend
CD4	BV650	1:200	GK1.5	BD Bioscience
IL-2	APC	1:100	JE56.5H4	Invitrogen
IL-17A	Alexa700	1:100	TC11-18H10.1	BioLegend
CD44	APC-Cy7	1:200	IM7	eBioscience
IL-5	PE	1:100	TRFKS	eBioscience
IFN-g	PE-Cy7	1:100	XMG1.2	eBioscience

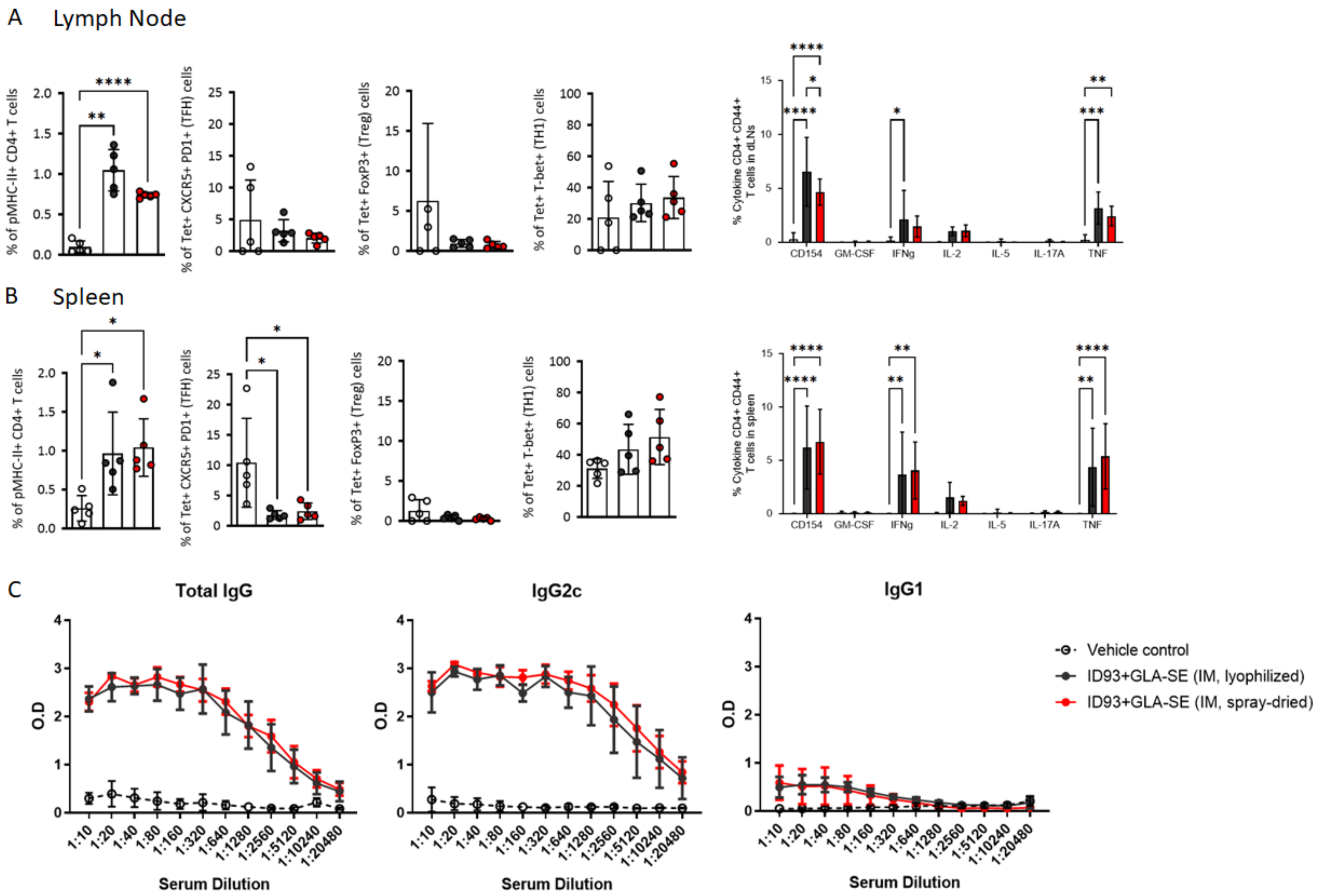

A Lymph Node
% of pMHC-II+ CD4+ T cells
% of Tet+ CXCR5+ PD1+ (TFH) cells
% of Tet+ FoxP3+ (Treg) cells
% of Tet+ T-bet+ (TH1) cells
% Cytokine CD4+ CD44+ T cells in dLNs
CD154
GM-CSF
IFNg
IL-2
IL-5
IL-17A
TNF
B Spleen
% of pMHC-II+ CD4+ T cells
% of Tet+ CXCR5+ PD1+ (TFH) cells
% of Tet+ FoxP3+ (Treg) cells
% of Tet+ T-bet+ (TH1) cells
% Cytokine CD4+ CD44+ T cells in spleen
CD154
GM-CSF
IFNg
IL-2
IL-5
IL-17A
TNF
C
Total IgG
IgG2c
IgG1
O.D
Serum Dilution
1:10 1:20 1:40 1:80 1:160 1:320 1:640 1:1280 1:2560 1:5120 1:10240 1:20480
Vehicle control
ID93+GLA-SE (IM, lyophilized)
ID93+GLA-SE (IM, spray-dried)

Supplementary Figure 1 (previous page). Reconstituted spray-dried ID93+GLA-SE administered intramuscular (IM) elicits highly similar immunogenicity profile as reconstituted lyophilized ID93+GLA-SE. C57BL/6 mice (n=5 females) were immunized once IM with reconstituted spray-dried ID93+GLA-SE, reconstituted lyophilized ID93+GLA-SE, or vehicle control (10% trehalose in 20 mM Tris buffer). Vaccinated groups received 0.4 µg ID93 and 1 µg GLA in 100 µL total volume (50 µL in each quadriceps). Draining lymph node, spleen, and serum were collected one week after immunization for immunogenicity analysis. (A) Percentage of antigen-specific CD4+ T cells and subsets (TFH, Treg, and TH1 cells) was assessed by tetramer staining and flow cytometry in draining lymph nodes. Percentage of antigen-specific cytokine-producing CD4+ T cells in the lymph node was measured by intracellular cytokine staining and flow cytometry. (B) Percentage of antigen-specific CD4+ T cells and subsets (TFH, Treg, and TH1 cells) was assessed by tetramer staining and flow cytometry in spleens. Percentage of antigen-specific cytokine-producing CD4+ T cells in the spleen was measured by intracellular cytokine staining and flow cytometry. (C) Antigen-specific serum antibody titers were assessed by ELISA. Statistical comparisons on tetramer stained CD4+ T cells were performed by one-way ANOVA or Welch's ANOVA with Tukey's or Dunnet's T3 correction, respectively, for multiple comparisons between selected groups. Statistical comparisons on cytokine-producing CD4+ T cells were performed by two-way ANOVA with Tukey's correction for multiple comparisons. Statistical comparisons on serum ELISAs are not shown. *$p<0.05$, **$p<0.01$, ***$p<0.001$, and ****$p<0.0001$.

A

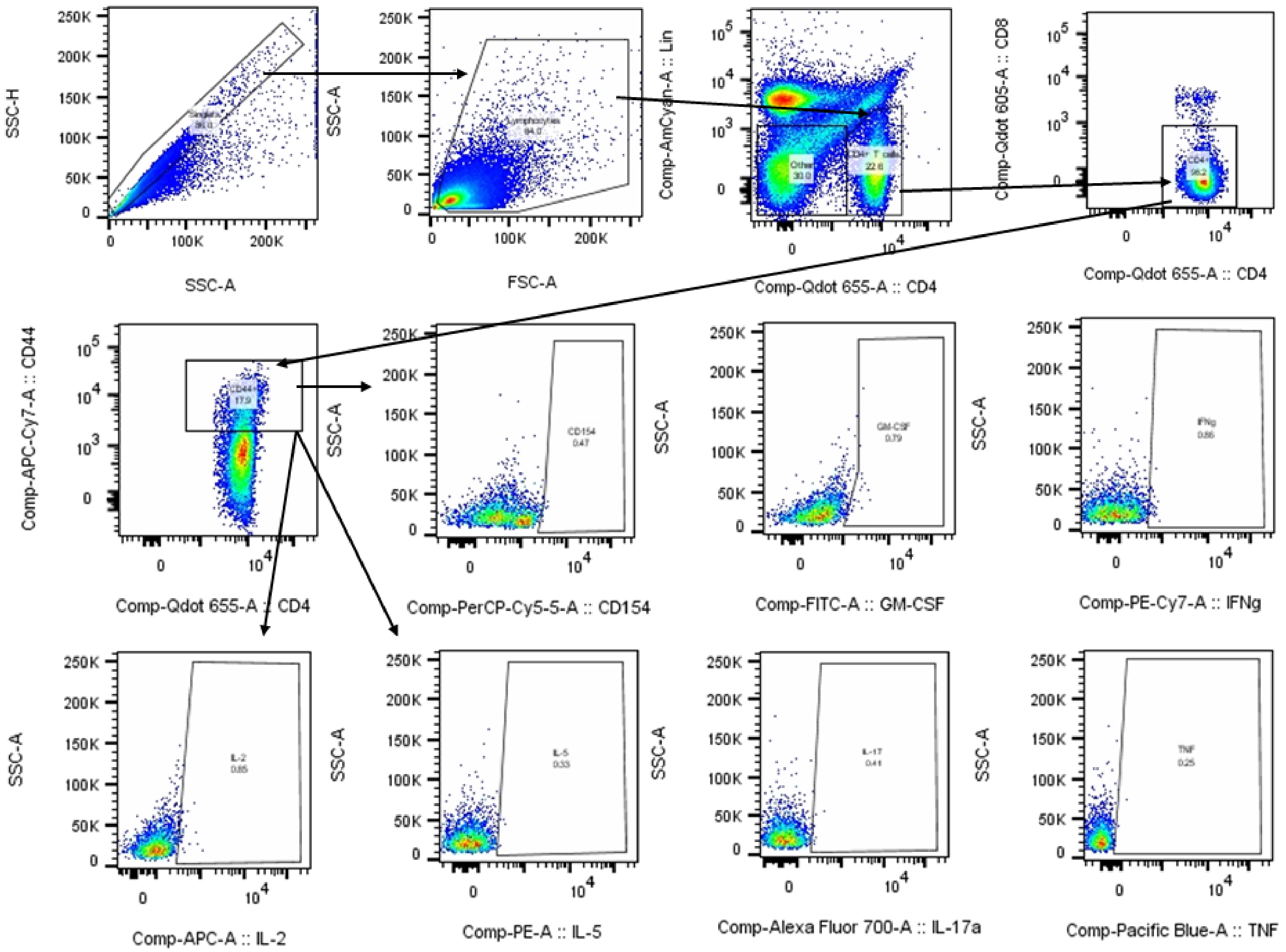
SSC-H
SSC-A
FSC-A
Comp-AmCyan-A :: Lin
Comp-Qdot 655-A :: CD4
Comp-Qdot 605-A :: CD8
Comp-APC-Cy7-A :: CD44
Comp-PerCP-Cy5-5-A :: CD154
Comp-FITC-A :: GM-CSF
Comp-PE-Cy7-A :: IFNg
Comp-APC-A :: IL-2
Comp-PE-A :: IL-5
Comp-Alexa Fluor 700-A :: IL-17a
Comp-Pacific Blue-A :: TNF

B

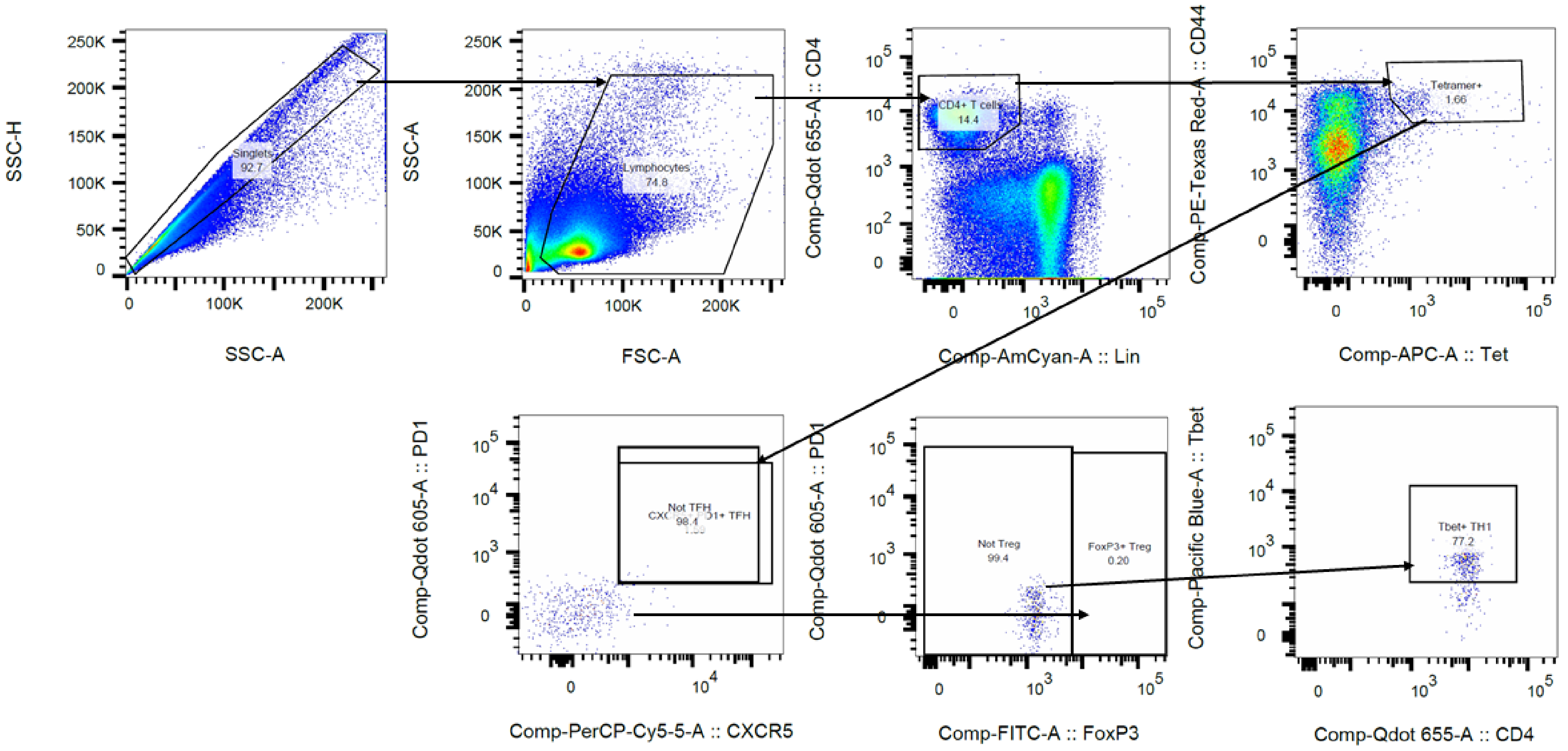

SSC-H
SSC-A
Singlets
92.7
Lymphocytes
74.8
FSC-A
Comp-Qdot 655-A :: CD4
CD4+ T cells
14.4
Comp-AmCyan-A :: Lin
Comp-PE-Texas Red-A :: CD44
Tetramer+
1.66
Comp-APC-A :: Tet
Comp-Qdot 605-A :: PD1
Not TFH
98.4
Comp-PerCP-Cy5-5-A :: CXCR5
Not Treg
99.4
FoxP3+ Treg
0.20
Comp-FITC-A :: FoxP3
Comp-Pacific Blue-A :: Tbet
Tbet+ TH1
77.2
Comp-Qdot 655-A :: CD4

C

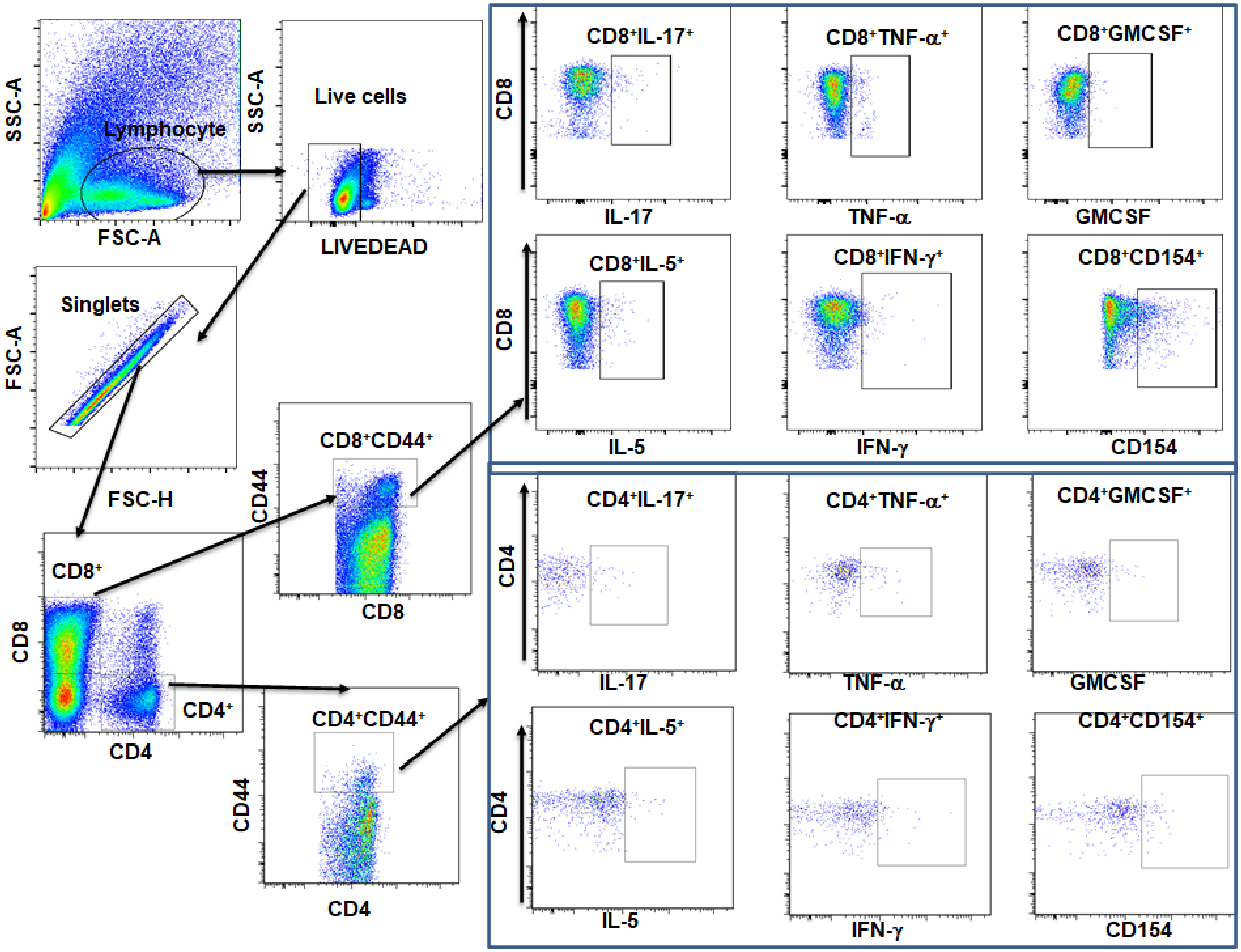
SSC-A
Lymphocyte
FSC-A
SSC-A
Live cells
LIVEDEAD
FSC-A
Singlets
FSC-H
CD8⁺
CD8
CD4⁺
CD4
CD8⁺CD44⁺
CD44
CD8
CD4⁺CD44⁺
CD44
CD4
CD8⁺IL-17⁺
CD8
IL-17
CD8⁺TNF-α⁺
TNF-α
CD8⁺GMCSF⁺
GMCSF
CD8⁺IL-5⁺
CD8
IL-5
CD8⁺IFN-γ⁺
IFN-γ
CD8⁺CD154⁺
CD154
CD4⁺IL-17⁺
CD4
IL-17
CD4⁺TNF-α⁺
TNF-α
CD4⁺GMCSF⁺
GMCSF
CD4⁺IL-5⁺
CD4
IL-5
CD4⁺IFN-γ⁺
IFN-γ
CD4⁺CD154⁺
CD154

Supplementary Figure 2 (previous 3 pages). Flow cytometry gating strategies for the immunogenicity experiments. (A) Intracellular cytokine staining flow cytometry gating strategy for the immunogenicity experiment in Supplementary Figure 1. (B) Tetramer staining flow cytometry gating strategy for immunogenicity experiment in Supplementary Figure 1. (C) Intracellular cytokine staining flow cytometry gating strategy for immunogenicity experiment in Manuscript Figure 5.

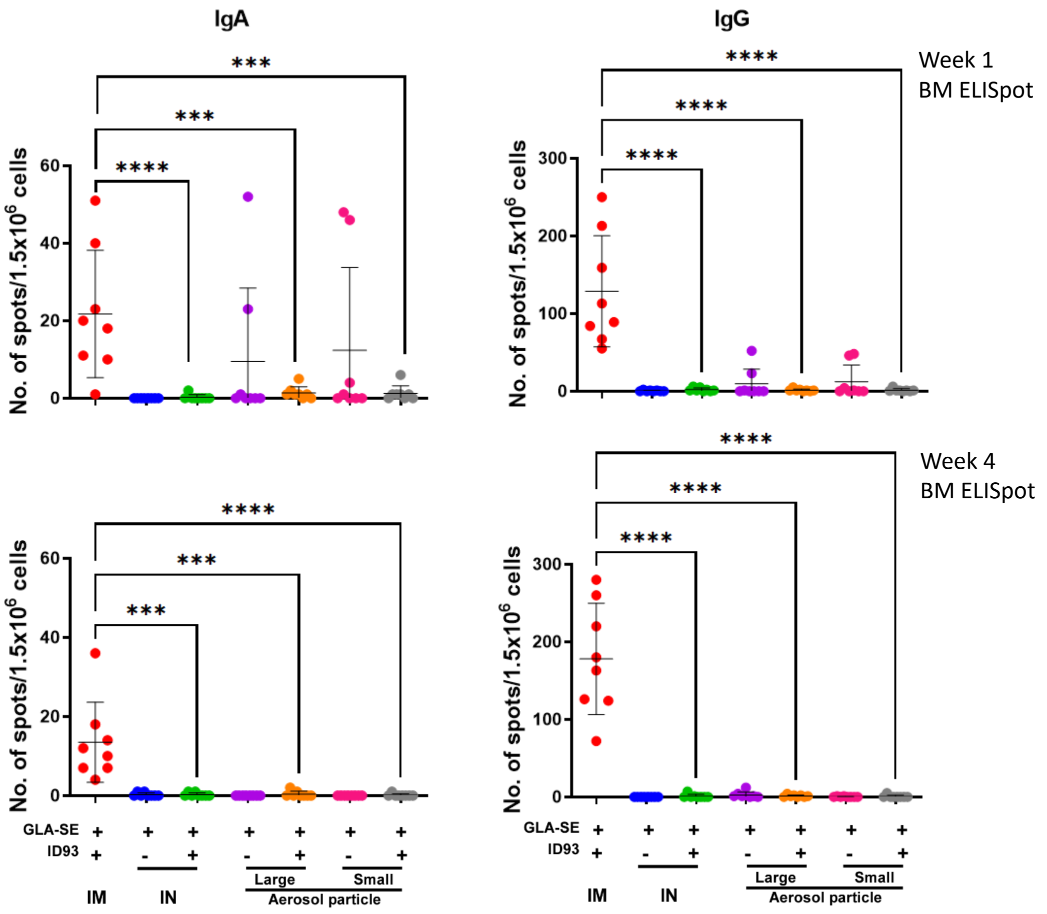

Supplementary Figure 3. IN or aerosol administration of ID93+GLA-SE does not induce long-lived B cell responses. B6 (n=8) mice were either vaccinated with ID93+GLA-SE or only GLA-SE through intramuscular (IM), intranasal (IN), or aerosol delivery of large or small dry powder particles at day 0 and day 21. One and four weeks after last booster, bone marrow cells were harvested, and IgG and IgA responses were determined by ID93-driven ELISpot assays. Data are presented as mean ± SD. Statistical comparisons were performed on log-transformed data (replacing zero values with 0.5) by one-way ANOVA or Welch's ANOVA with Sidak's or Dunnet's T3 correction, respectively, for multiple comparisons between selected groups. The Kruskal-Wallis non-parametric test with Dunn's correction for multiple comparisons was used when multiple comparisons were not possible with Welch's ANOVA due to limitations of the latter test when one experimental group had constant values.

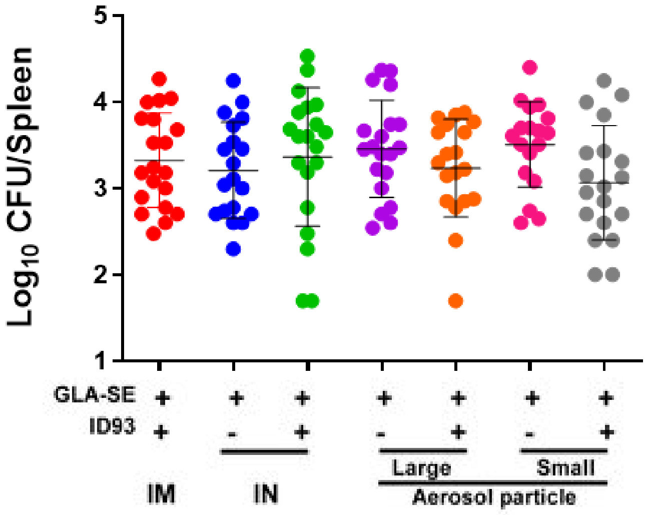

Supplementary Figure 4. Delivery of spray-dried ID93+GLA-SE administered by the indicated routes did not significantly reduce *Mtb* CFU titers in the spleen compared to adjuvant alone. B6 mice (n=18-20) were vaccinated with ID93+GLA-SE or only GLA-SE through reconstituted liquid intramuscular (IM), reconstituted liquid intranasal (IN), or aerosol delivery of large or small dry powder particles at day 0 and day 21. All groups of B6 mice were rested for 4 weeks after which mice were challenged with *Mtb* H37Rv (100 CFU). *Mtb* CFU was determined at 4 weeks post-infection. Data presented are combined results from two identical experiments, showing mean ± SD. Statistical comparison was performed by one-way ANOVA with Sidak's correction for multiple comparisons between selected groups.
